# Control of Neural Daughter Cell Proliferation by Multi-level Notch/Su(H)/E(spl)-HLH Signaling

**DOI:** 10.1371/journal.pgen.1005984

**Published:** 2016-04-12

**Authors:** Caroline Bivik, Ryan B. MacDonald, Erika Gunnar, Khalil Mazouni, Francois Schweisguth, Stefan Thor

**Affiliations:** 1 Department of Clinical and Experimental Medicine, Linkoping University, Linkoping, Sweden; 2 Institut Pasteur, Paris, France; 3 CNRS, URA2578, Paris, France; Washington University Medical School, UNITED STATES

## Abstract

The Notch pathway controls proliferation during development and in adulthood, and is frequently affected in many disorders. However, the genetic sensitivity and multi-layered transcriptional properties of the Notch pathway has made its molecular decoding challenging. Here, we address the complexity of Notch signaling with respect to proliferation, using the developing *Drosophila* CNS as model. We find that a Notch/Su(H)/E(spl)-HLH cascade specifically controls daughter, but not progenitor proliferation. Additionally, we find that different *E(spl)-HLH* genes are required in different neuroblast lineages. The Notch/Su(H)/E(spl)-HLH cascade alters daughter proliferation by regulating four key cell cycle factors: Cyclin E, String/Cdc25, E2f and Dacapo (mammalian p21^CIP1^/p27^KIP1^/p57^Kip2^). ChIP and DamID analysis of Su(H) and E(spl)-HLH indicates direct transcriptional regulation of the cell cycle genes, and of the Notch pathway itself. These results point to a multi-level signaling model and may help shed light on the dichotomous proliferative role of Notch signaling in many other systems.

## Author Summary

Communication between cells is critical for controlling proliferation, and the Notch signal transduction pathway plays a well-established and evolutionary conserved role during these processes. However, in spite of numerous studies of this pathway over the years, the genetic sensitivity of the pathway, combined with complexity in the nuclear response to Notch activation, has often precluded an in-depth molecular understanding of the pathway. In addition, findings in many systems point to both anti- and pro-proliferative roles of Notch signaling. Here, we use a number of novel genetic strains–mutants and misexpression transgenes–and focus on a particular role of the pathway; daughter cell proliferation in the embryonic *Drosophila* central nervous system. Combined with genome-wide chromatin binding assays, we are able to decode the pathway and identify both the nuclear effectors downstream Notch, as well the key cell cycle genes involved. We find that Notch activity is gated by a process of direct and indirect transcriptional output, which acts to balance the proliferation decision with high fidelity. These findings shed light on the dichotomous nature of Notch signaling with respect to proliferation control and may point to widely used aspects of the pathway.

## Introduction

The Notch signal transduction pathway plays a central role during animal development, and is also critical for tissue homeostasis during adulthood [1]. Notch signaling typically acts as a short-range, cell-cell communication system, which can trigger a multitude of cellular responses, including proliferation, differentiation and programmed cell death. The outcome of Notch activation is highly context-dependent, and with respect to e.g., proliferation, Notch can act both as an anti- and pro-proliferative regulator [2].

The dynamic response of the genome to Notch receptor activation is multi-faceted [3, 4]. The immediate response involves a tripartite protein complex consisting of the intracellular domain of Notch (NICD), the DNA-binding factor Su(H) (mammalian RBPJ) and the co-factor Mastermind (mammalian Maml1/3) [5, 6]. In *Drosophila*, main direct targets of the NICD-Mam-Su(H) activator complex are the genes of the *Enhancer-of-split* Complex (*E(spl)-C*); founding members of the HES gene family of bHLH transcriptional repressors [7–9]. A delayed response to Notch activation therefore likely involves the repression of secondary target genes by the E(spl)-HLH factors. During early neurogenesis, these E(spl)-HLH factors act by antagonizing the activity and expression of the proneural bHLH factors [10]. However, the full repertoire of HES/E(spl)-HLH targets remains largely unknown. Additionally, *E(spl)-HLH* gene activation by NICD-Su(H)-Mam is context-dependent i.e., different *E(spl)-HLH* genes are activated in response to Notch in different tissues [11]. Therefore, the precise flow of events from receptor cleavage to diverse target gene regulation is often unclear: which specific *E(spl)-HLH* genes are activated, which other target genes are regulated, and at what level(s)? For instance, while Notch signaling is known to regulate cell cycle genes [12], it is unclear whether this regulation is direct via NICD-Mam-Su(H), or indirect via the *E(spl)-HLH* factors; chiefly because the genome-wide binding profiles of *E(spl)-HLH* factors have not been addressed. Finally, whether differences in E(spl)-HLH expression and function contribute to the cell-specific response to Notch receptor activation remains completely unknown, primarily because extensive genetic redundancy has precluded the identification of single-gene mutations and functions for any one of these genes [13–16].

**Fig 1:**
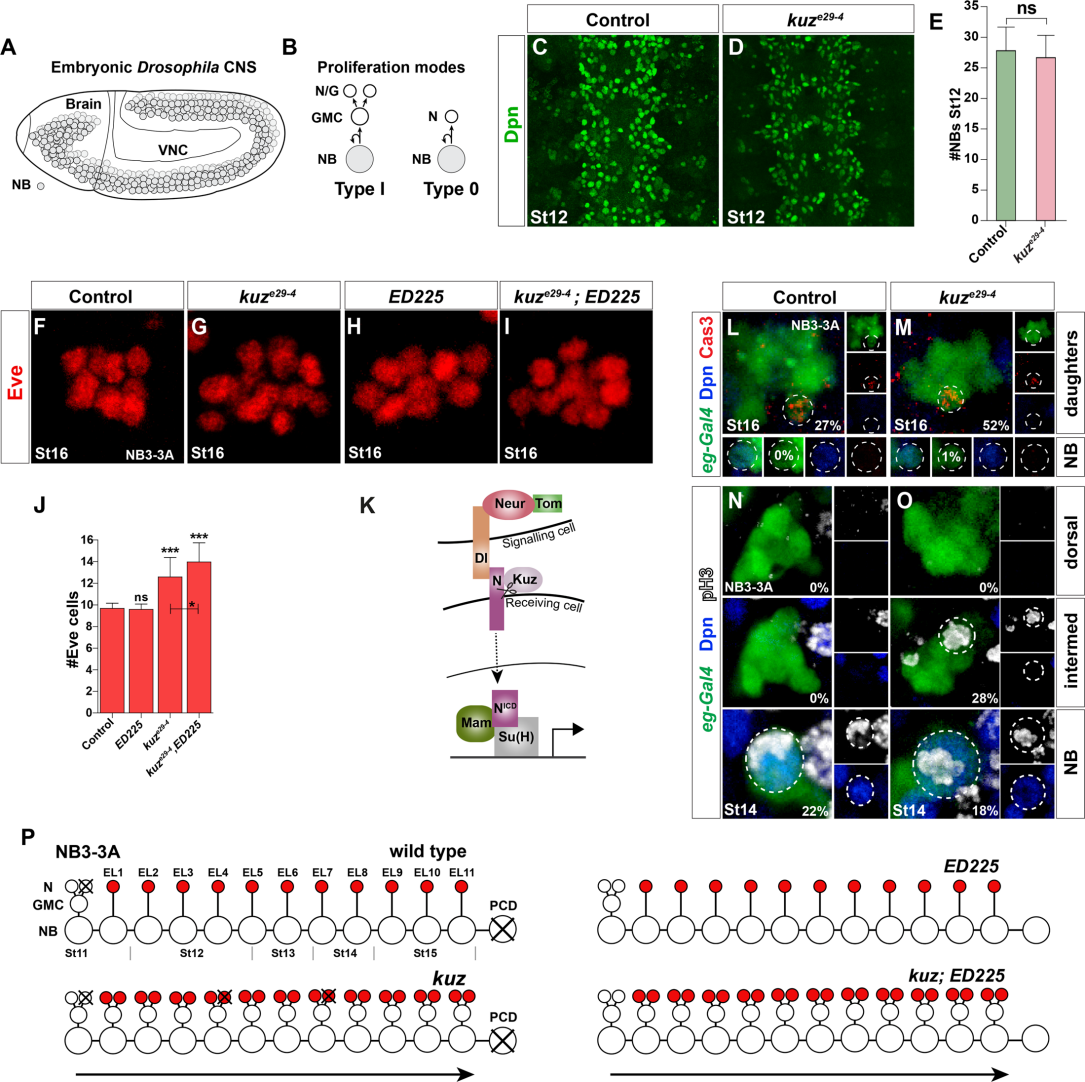
Notch pathway controls Type I>0 daughter proliferation switch in NB3-3A independently of programmed cell death. (A) Embryonic *Drosophila* CNS at St11; NBs outlined. (B) NBs can divide by Type I or Type 0 mode, and many lineages display a Type I>0 switch [23]. (C-E) *kuz*^*e29-4*^ mutants have normal NB numbers (n = 40 T2-A2 segments; Student’s two-tailed T-test; +/-SD). (F-I) The Type 0 cells in the NB3-3A lineage can be visualized by Eve expression. (F) In control at St16, an average of 9.5 Eve cells is observed. (H) *ED225* programmed cell death (PCD) mutants show similar numbers (E). (I) *kuz*^*e29-4*^;*ED225* double mutants show additional Eve cells, when compared to both *kuz*^*e29-4*^ and *ED225* single mutants. (J) Quantification of Eve cell number (* p ≤0.05, ** p≤0.01, *** p≤0.001; n≥60 clusters; Kruskal-Wallis, Dunn’s posthoc; +/-SD). (K) Canonical Notch pathway. (L-M) In line with the extra Eve cells found in the *kuz*^*e29-4*^;*ED225* double mutants, staining for apoptotic cells, using anti-cleaved Caspase3 as read-out, revealed increased PCD in *kuz*^*e29-4*^. There was however no sign of premature cell death of the NB itself. (N-O) NB and daughter proliferation analysis in NB3-3A. (N) As previously described [23], in control after St11 we exclusively detected cell division of the NB itself (Dpn+) (0% daughter divisions; n≥88 lineages). (O) In contrast, in *kuz*^*e29-4*^, we readily detected dividing daughter cells (28% daughter divisions; n≥65 lineages; dorsal and intermed refers to dorsal and intermediate confocal layers, respectively). (Q) In wild type, the NB3-3A undergoes one Type I and 11 Type 0 divisions. The NB exits cell cycle at St15, and undergoes PCD at St17 [23]. There is limited PCD, in the early parts of the lineage. Additional Eve cells in *kuz* result from a failure in the Type I>0 switch. In *kuz* mutants, some of the aberrantly generated cells are removed by PCD. In *ED225* mutants, the NB does not undergo PCD, but does not progress further [23]. In *kuz*,*ED225* mutants, aberrantly generated cells survive, hence increasing the Eve cell numbers beyond that observed in *kuz* alone.

Here, we address the connection between Notch signaling and proliferation control using the *Drosophila* embryonic CNS as model. The CNS is established by some 1,200 neuroblasts (NBs) that delaminate from the neurogenic ectoderm (Fig 1A) [17–20]. NBs divide asymmetrically to self-renew and produce daughter cells with a more limited proliferation potential [21]. For the majority of NBs, early-born daughter cells divide once, to generate two neurons/glia; denoted Type I proliferation mode [22] (Fig 1B). We recently demonstrated that many, perhaps all, NBs undergo a programmed proliferative switch, to generate daughters that directly differentiate into neurons; Type 0 proliferation mode [23](Fig 1B). This Type I>0 proliferation switch requires critical input from a few key cell cycle genes, including *Cyclin E* (*CycE*), *string* (*stg*; mammalian cdc25), *E2f* and *dacapo* (*dap*; p21^CIP1^/p27^KIP1^/p57^Kip2^).

In this study, we find that Notch/E(spl)-HLH signaling is globally required to regulate the Type I>0 switch. To dissect the Notch downstream events and the role of the different *E(spl)-HLH* genes, we utilized TILLING and CRISPR/Cas9 mutagenesis, as well as BAC recombineering, to generate novel individual mutants for all seven *E(spl)-HLH* genes. Strikingly, in spite of their reported genetic redundancy, we find that, when placed over a genomic deletion removing all seven genes, individual *E(spl)-HLH* mutations can significantly affect the Type I>0 daughter proliferation switch. Intriguingly, different *E(spl)-HLH* genes affect the switch in different NB lineages. With respect to cell cycle components, Notch signaling regulates several key cell cycle proteins, including CycE, E2f, Stg and Dap. Moreover, ChIP-seq and DamID-seq demonstrates binding of Su(H), E(spl)m5-HLH and E(spl)m8-HLH to *E(spl)-C*, *CycE*, *stg*, *E2f* and *dap*. These results help resolve the Notch pathway with respect to the Type I>0 switch, by identifying the main Notch components, the critical downstream targets, as well as the molecular and genetic interactions involved. We propose an intriguing multi-levelNotch signaling cassette involved in the Type I>0 daughter proliferation switch, where primary-level Notch signaling results in activation of *E(spl)-HLH* and cell cycle genes, and second-level Notch signaling results in *E(spl)-HLH* repressing a partly overlapping set of cell cycle genes. This multi-levelmode of Notch signaling may help ensure precise timing and fidelity of the Type I>0 switch, and may shed light upon the sensitivity and dynamics of Notch signaling, as well as its dichotomous nature with respect to proliferation control, in many other systems.

## Results

### The Notch Pathway Controls the Type I>0 Daughter Cell Proliferation Switch

The embryonic *Drosophila* CNS can be subdivided into the brain and the ventral nerve cord (VNC); here we focus on the VNC. Each embryonic VNC hemisegment contains ~30 lateral NBs [24, 25], most, if not all of which commence neurogenesis by proliferating in the Type I mode [22]. Subsequently many, perhaps all, switch to the Type 0 mode (Fig 1B)[23]. We previously used two model lineages to study the Type I>0 switch; NB5-6T and NB3-3A, both of which can be uniquely identified by transgenic reporters and a number of markers. NB5-6T undergoes nine rounds of Type I proliferation, followed by five rounds of Type 0 proliferation, while NB3-3A undergoes one Type I round, followed by 11 Type 0 rounds (Figs 1Q and S1G)[23, 26, 27]. The last four Type 0 neurons in NB5-6T are denoted Apterous (Ap) neurons [26] and can be identified by Eyes absent (Eya)[28]. Similarly, the Type 0 neurons in NB3-3A can be identified by Even-skipped (Eve) [19, 26, 29].

The Notch pathway is critical for the Type I>0 switch in NB5-6T [27](S1A–S1F Fig). We find similar effects in NB3-3A (Fig 1F–1J). Early and strong Notch pathway perturbation results in a failure of lateral inhibition, and as an effect thereof the generation of supernumerary NBs [30, 31]. However, *kuzbanian* (*kuz*) mutants, presumably due to the maternal expression of *kuz*, showed extra Ap neurons without extra NB5-6T [27], and we also find extra Eve neurons without extra NB3-3A neuroblasts (Fig 1N and 1O). In line with these findings, we found no change in overall NB numbers in *kuz*^*e29-4*^ (Fig 1C–1E). In spite of *kuz*^*e29-4*^ being a likely null allele [32], we did not observe a full penetrance of the phenotype i.e., with all Type 0 daughters converting to Type I, again likely due to the maternal expression of *kuz*. Addressing other Notch signaling components during the Type I>0 switch revealed roles for *Su(H)*, *Tom* and *neuralized* (*neur*), as well as the *Delta* but not *Serrate* ligand [27] (S1C–S1F Fig). Hence, canonical Notch signaling is involved in the Type I>0 switch in both NB5-6T and NB3-3A (Figs 1Q and S1G).

**Fig 2:**
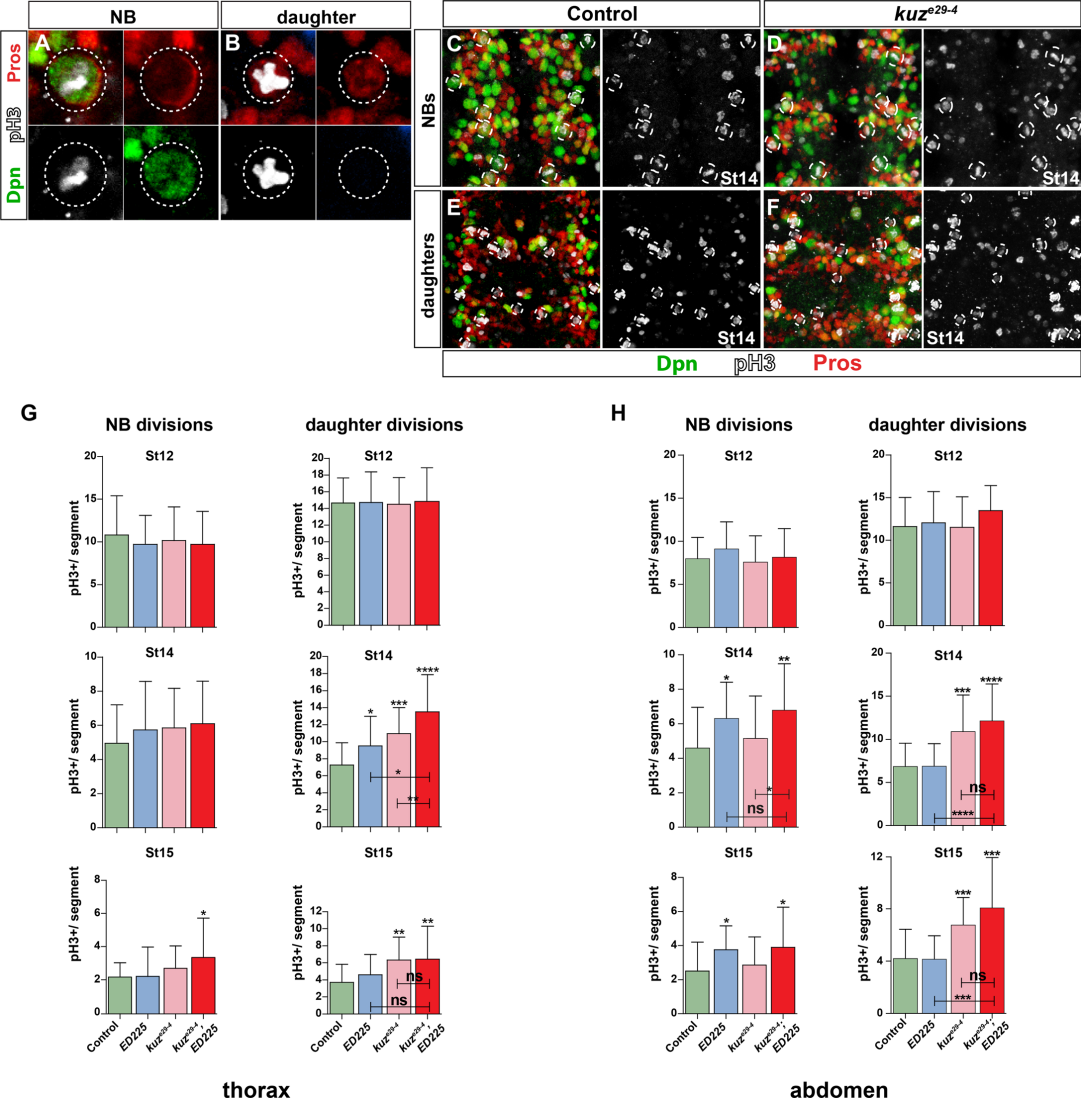
Notch pathway controls Type I>0 daughter proliferation switch globally. (A) Dividing NB, identified by Dpn, phosphorylated-HistoneH3 (pH3), and cortical asymmetric Pros. (B) Dividing daughter cell (GMC), recognized by nuclear Pros and pH3, and lack of Dpn. (C-F) Dividing NBs and daughters in T2-T3. *kuz*^*e29-4*^ shows increase in dividing daughters when compared to control. (G-H) Quantification of dividing cells in T2-T3 and A1-A2, in control, *kuz*^*e29-4*^, *ED225*, and *kuz*^*e29-4*^;*ED225* double mutants. In the thorax, NB divisions are only affected in *kuz*^*e29-4*^;*ED225* double mutants at St15. Daughter divisions are however increased, particularly in *kuz*^*e29-4*^ and *kuz*^*e29-4*^; *ED225* double mutants, both at St14 and St15. In the abdomen, NB divisions are increased in *ED225* and *kuz*^*e29-4*^;*ED225* double mutants, at St14 and St15. Daughter divisions are also increased, particularly in *kuz*^*e29-4*^ and *kuz*^*e29-4*^; *ED225* double mutants (* p≤0.05, ** p≤0.01, *** p≤0.001; Student’s two-tailed T-test; +/-SD; n≥20 segments).

Next, we analyzed global proliferation pattern in both the abdominal and thoracic VNC. To this end we stained VNCs with Deadpan (Dpn), Prospero (Pros) and phosphorylated histone H3 (pH3), allowing us to discriminate between dividing NBs (Dpn+ and cortical/asymmetric Pros) and dividing daughters (Dpn-negative and cellular Pros; [23]) (Fig 2A and 2B). We analyzed NB and daughter proliferation at three stages, in thoracic T2-T3 and abdominal A1-A2 segments. We did not find any global NB proliferation effects in *kuz*^*e29-4*^ (Fig 2C and 2D and 2G and 2H). In contrast, *kuz*^*e29-4*^ showed increase in dividing daughter cells, in both thorax and abdomen, at both St14 and St15 (Fig 2E and 2F and 2G and 2H).

Notch signaling has been shown to be involved in Programmed Cell Death (PCD) in the VNC, specifically of postmitotic cells [33]. This raised the possibility that reduced daughter proliferation observed in Notch pathway mutants may result from reduced PCD of Type I daughters, rather than conversion of Type 0 daughter to Type I. To address this issue, proliferation analysis was also conducted in PCD mutants (*Df(3R)ED225*), as well as in *kuz*, *ED225* double mutants, in NB3-3A, NB5-6T and globally. We found minimal global proliferation effects in *ED225*, apparent only in thoracic daughters at St14 and abdominal NBs at St14 and St15 (Fig 2G and 2H). *kuz*^*e29-4*^; *ED225* double mutants showed increased NB proliferation similar to *ED225* single mutants, and increased daughter proliferation similar to *kuz*^*e29-4*^ single mutants (Fig 2G and 2H). Similar effect of *ED225* was observed also in NB3-3A and NB5-6T (Figs 1H–1J and S1E and S1F). These results are in line with previous published findings on PCD and lineage progression, and demonstrates that Notch signaling does not trigger the Type I>0 switch by merely triggering PCD (see S1H Fig and S1 Fig legend for details and references).

In summary, canonical Notch pathway signaling is globally involved in the Type I>0 daughter proliferation switch in the VNC, but does not control this switch via PCD.

### Differential Function of Distinct *E(spl)* Genes in the Type I>0 Switch

To begin addressing the downstream events involved in the Notch-mediated Type I>0 switch, we focused on the *Enhancer-of-split* complex (*E(spl)*) effectors in the Notch pathway. This complex contains seven *HES/E(spl)-HLH* genes, displaying well-known genetic redundancy [13–16]; to date, no single gene loss-of-function phenotype has been described.

We first analyzed a series of deletions in the regions, and found strong effects on Ap cell numbers (Eya+) in NB5-6T (S2A–S2F Fig). Extra Ap neurons observed in *E(spl)* complex mutants arose from both failure of NB selection and Type I>0 switch (S2E Fig), with weaker genotypes only affecting the switch and stronger genotypes affecting both the switch and NB selection i.e., lateral inhibition (S2F and S2G Fig). Together, these results point to a role in the VNC for: *m7* and/or *m8*; *m3* and/or *m5*; *mδ*, *mγ* and/or *mβ*; as well as *gro*.

**Fig 3:**
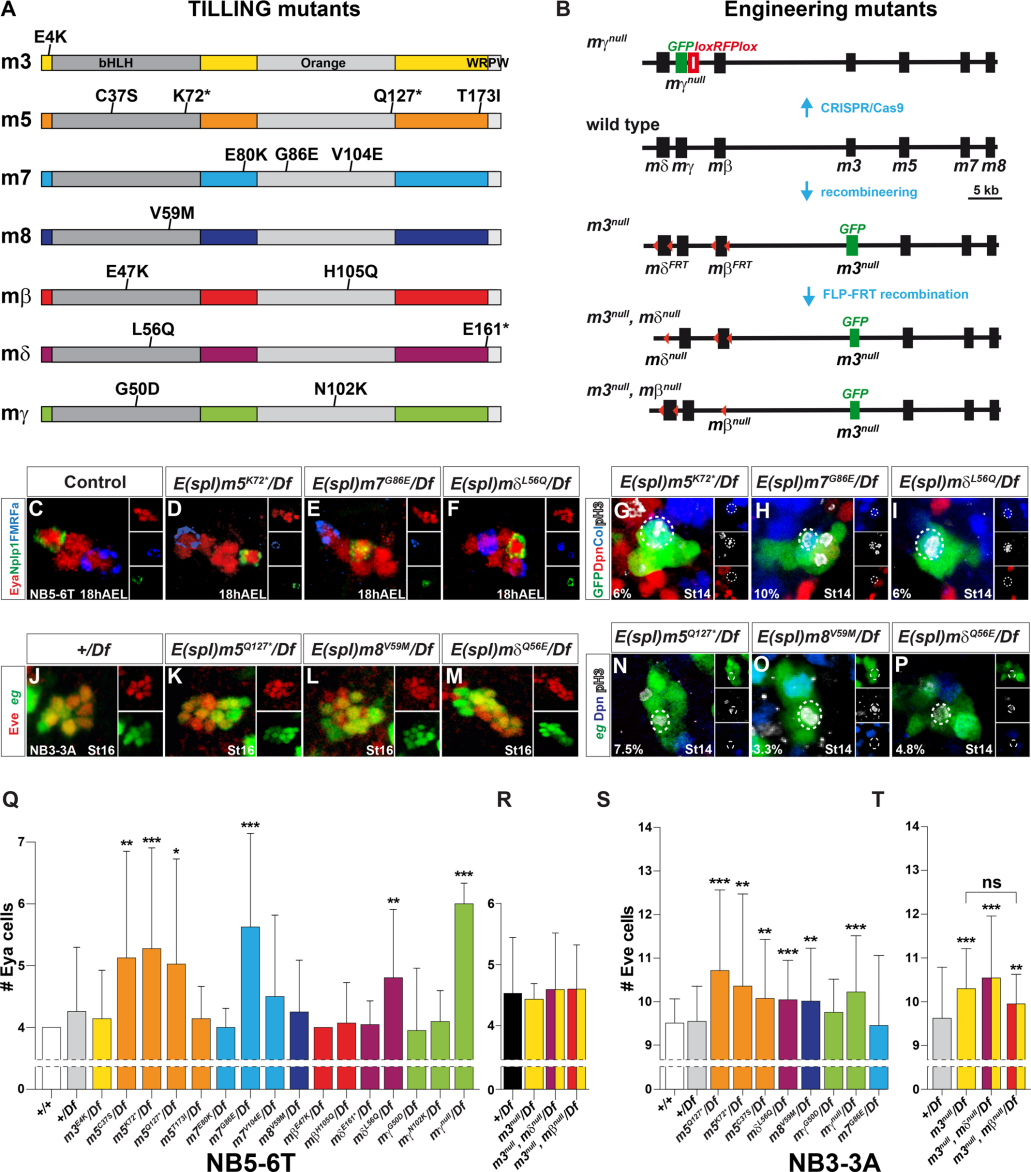
Single and differential *E(spl)-HLH* gene function in neuroblasts. (A-B) Outline of *E(spl)-HLH* TILLING, CRISPR/Cas9 and recombineering mutants. (C) Control, showing the four Ap cells (Eya+) in T2-T3. (D-F) Extra Ap cells, and (G-I) aberrantly dividing NB5-6T daughter cells; in *m5*^K72*^, *m7*^*G86E*^ and *mδ*^*Q56E*^, when placed over deletion *Df(3R)BSC751* (G-I; percentages denote NB5-6T lineages with a dividing daughter cell). (J-M) Extra Eve cells, and (N-P) aberrantly dividing NB3-3A daughter cells; in *m5*^Q127*^, *m8*^*V59M*^ and *mδ*^*Q56E*^, when placed over deletion *Df(3R)BSC751* (percentages denote NB3-3A lineages with a dividing daughter cell). (Q-T) Quantification of phenotypes. Four *E(spl)-HLH* genes show significant effects in NB5-6T, while five show effects in NB3-3A. Notably, *m7* only affects NB5-6T, while *m3* and *m8* only affects NB3-3A (* p≤0.05, ** p≤0.01, *** p≤0.001; +/-SD; Kruskal-Wallis, Dunn’s posthoc; n≥22 lineages). TILLING alleles and the CRISPR/Cas9 *mγ*^*null*^ allele were placed over deletion *Df(3R)BSC751*. The recombineering alleles; *m3*^*null*^, *mδ*^*null*^ and *mβ*^*null*^, were deleted in a BAC, inserted on chromosome 2 (51D) and crossed into a *Df(3R)gro32*.*2*, *P-gro/*(*E(spl)-C*^*Δmδ-m6*^ genetic background. Control was *Df(3R)gro32*.*2*, *P-gro*/+.

To resolve the individual roles of the seven *E(spl)-HLH* genes, TILLING was performed to identify EMS-induced mutations from a genome-wide mutagenesis project. A number of mutations in all seven genes were identified, out of which 15 nonsense and missense mutations, predicted to affect protein function, were chosen for further study (Fig 3A and S1 Table). In addition, we utilized recombineering to generate complete deletions of three genes, as single or double mutants: *m3*^*null*^; *m3*^*null*^,*mδ*^*null*^; and *m3*^*null*^,*mβ*^*null*^ (Fig 3B). Finally, *mγ*^*null*^ was engineered by CRISPR/Cas9 mediated deletion of the coding region of the gene (Fig 3B). TILLING alleles, as well as the CRISPR/Cas9 *mγ*^*null*^ allele, were tested over a deletion for the region (*Df(3R)BSC751*) which removes all seven *E(spl)* genes and *gro* (S2A Fig). The recombineering alleles (*m3*^*null*^, *mδ*^*null*^ and *mβ*^*null*^) were deleted in a BAC, inserted on chromosome 2 (*51D*), and crossed into a *Df(3R)gro32*.*2*, *P-gro/*(*E(spl)-C*^*Δmδ-m6*^ genetic background. We initially focused on NB5-6T, and found increase in Ap cell numbers for four genes; *mδ* (*mδ*^*L56Q*^), *m5* (*m5*^*C37S*^, *m5*^K72*^, *m5*^Q127*^), *m7* (*m7*^*G86E*^) and *mγ* (*mγ*^*null*^) (Fig 3C–3F and 3Q). None of the three null alleles for *m3*, *mδ* or *mβ* showed increase in Ap cell numbers (Fig 3R). The reason *mδ*^*L56Q*^ showed effect while *mδ*^*null*^ did not may be due to that *mδ*^*L56Q*^ was tested in a *gro* heterozygous background, whereas *mδ*^*null*^ was in a wild type background with respect to *gro*. The *m8*^*V59M*^ mutant did not show any effect (Fig 3Q). Analysis of the NB5-6T lineage revealed that extra Ap neurons resulted from defects in the Type I>0 switch, as evident by pH3+, late-born (Type 0) daughters in the lineage (Fig 3G–3I). Underscoring the redundant nature of the region, homozygous *m5* mutants did not show increase in Ap cell numbers (S3A Fig).

To address if *E(spl)-HLH* gene involvement may vary between NBs, we next analyzed the *E(spl)-HLH* mutants for effects upon NB3-3A development. Similar to our findings on NB5-6T, the *m5*, *mδ* and *mγ* mutants displayed increase also in Eve cells (Fig 3J–3M and 3S and 3T). However, in contrast to NB5-6T, we did not find effects in *m7* mutants in NB3-3A (Fig 3S). Instead, while the *m8*^*V59M*^ mutant did not affect NB5-6T, it did show effect on NB3-3A (Fig 3L and 3S). In line with this finding, we observed expression of an *m8-GFP* reporter in the NB3-3A neuroblast (S3B Fig). Moreover, the recombineering alleles revealed effects for *m3*^*null*^, and as expected for *m3*^*null*^,*mδ*^*null*^ (Fig 3T). However, in *m3*^*null*^,*mβ*^*null*^ double-mutants Eve cell numbers were not increased beyond that observed in *m3*^*null*^ alone (Fig 3T). Together with lack of phenotype in the *mβ* TILLING alleles, this argues against any role for *mβ*. Analysis of pH3-positive cells in the NB3-3T lineage revealed that extra Eve neurons resulted from defects in the Type I>0 switch, evident by pH3-positive, late-born (Type 0) daughters in the lineage (Fig 3N–3P).

These studies demonstrate that in spite of redundancy between the *E(spl)* genes in relation to other Notch functions, with respect to the Type I>0 switch we observe weak but significant effects in single gene mutants for six of the seven *E(spl)-HLH* genes, revealed when placed over a deficiency removing the entire *E(spl)* region. Strikingly, we furthermore find evidence for selective utilization of different *E(spl)* genes in different NBs, with *m3* and *m8* only acting in NB3-3A and *m7* in NB5-6T.

### *kuz* and *E(spl)-HLH* Interact with the Cell Cycle Genes *dacapo* and *Cyclin E*

Next we aimed to identify the downstream targets of Notch/E(spl)-HLH signaling with regards to the Type I>0 switch. While the proneural genes are well-known to be regulated by Notch [34, 35], our studies indicate that they are not the key targets of Notch signaling in the switch (S4A–S4F Fig).

**Fig 4:**
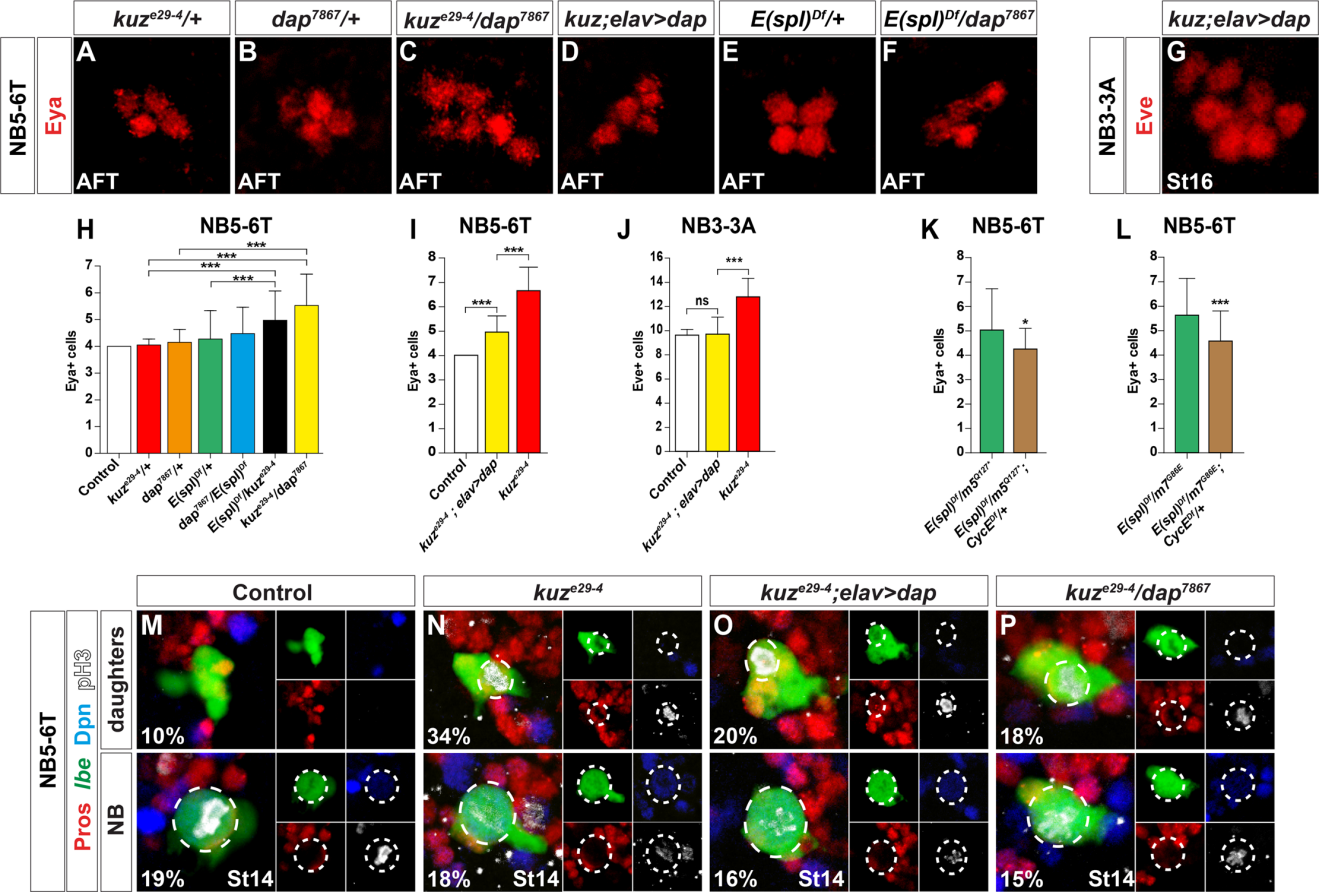
The Notch pathway interacts with *dap* and *CycE*. (A-F) Eya cells in the NB5-6T lineage, and (G) Eve cells in the NB3-3A lineage. (A-B) In *kuz*^*e29-4*^*/+* or *dap*^*7867*^*/+* there is no effect on Ap cell (Eya+) numbers. (C) In contrast, *kuz*^*e29-4*^*/dap*^*7867*^ trans-heterozyogotes display increased Ap cell numbers. (D, G) Expression of *dap* in homozygous *kuz*^*e29-4*^ mutants, driven by *elav-Gal4*, reduces the number of Ap and Eve cells. (E-F) Neither *E(spl)Df/+* heterozygotes (*Df(3R)BSC751/+*) nor *dap*^*7867*^/+;*E(spl)Df/+* trans-heterozygotes show any effect on Ap numbers. (H-L) Quantification of Ap and Eve cell numbers (* p≤0.05, ** p≤0.01, *** p≤0.001; +/-SD; n≥40 lineages). (K-L) Reducing *CycE* gene dosage by half (*CycE*^*Df*^ *= Df(2L)BSC255/+*), significantly suppresses the number of Ap cells in *m5/ Df(3R)BSC751*and *m7/ Df(3R)BSC751* mutants. (M-P) Dividing NBs (bottom) and daughter cells (top) in the NB5-6T lineage. (M) In control, NB divisions are observed in 19% of lineages, while only 10% of lineages show dividing daughters. (N) In *kuz*^*e29-4*^, NB divisions are similar to control, while daughter divisions are increased to 34%. (O) Expression of *dap* in a *kuz*^*e29-4*^ mutant background does not apparently affect the number of NB divisions, but does reduce daughter divisions down from 34% to 20%. (P) In *kuz*^*e29-4*^*/dap*^*7867*^ trans-heterozyogotes, NB divisions are in line with control (15%), while daughter divisions are at 18%.

We recently demonstrated that proliferation control in the developing *Drosophila* VNC requires four key cell cycle factors: Cyclin E (CycE), E2f, String (Stg; mammalian Cdc25) and Dacapo (Dap; mammalian p21^CIP1^/p27^KIP1^/p57^Kip2^); mutation and/or misexpression of these cell cycle genes affects the Type I>0 daughter proliferation switch [23, 36]. These findings prompted us to test for genetic interactions and cross-rescue between Notch and these genes, again using the Ap neurons in NB5-6T (Eya+ cells) and the Eve+ neurons in NB3-3A as readouts for a defective Type I>0 switch. First, we tested for trans-heterozygotic interaction between *kuz* and *dap*, and strikingly, noted an increase in both Ap and Eve neurons (Figs 4A–4C and 4H and S5A). We also noted genetic interaction between *kuz* and *E(spl)-C* (Fig 4H). Second, we attempted to rescue *kuz* by transgenic expression of *dap*, and observed suppression of the number of Ap and Eve neurons (Fig 4D and 4G and 4I and 4J). These genetic interaction and cross-rescue effects were due to specific effects upon the Type I>0 switch, as evident by the alterations in daughter but not NB divisions in the NB5-6T lineage at St13 (Fig 4M–4P). In contrast to *kuz/dap* and *kuz/E(spl)-C* interactions, we noted no interaction between *dap* and a *E(spl)* complex deletion (Fig 4E and 4F and 4H). Third, we attempted to suppress the increase of Ap neurons in *m5* and *m7* hemizygous mutants by reducing *CycE* gene dosage, and indeed observed reduction of supernumerary Ap neurons (Fig 4K and 4L).

We conclude that, with respect to the Type I>0 daughter proliferation switch, there is genetic interaction between the Notch/E(spl)-HLH pathway and the *dap* and *CycE* cell cycle genes.

### Misexpression of *E(spl)m8-HLH* Triggers Premature Type I>0 Daughter Proliferation Switch

A number of gain-of-function studies have demonstrated strong effects when expressing the Notch-Intracellular Domain truncation (NICD) [37]. To address the sufficiency of Notch signaling to trigger the Type I>0 switch, we therefore misexpressed NICD using the *insc-Gal4* driver, a driver expressed by most if not all NBs from St11 and onwards. We analyzed NB and daughter proliferation in both the thorax and abdomen, at two different stages. In line with the selective role for Notch signaling in controlling daughter but not NB proliferation, we did not observe any effect on NB proliferation at any stage, neither in thorax nor abdomen (S5B Fig). We did however observe significant reduction of daughter proliferation, evident in both thorax and abdomen at St12 (S5B Fig). We thus find that NICD can trigger the Type I>0 switch.

**Fig 5:**
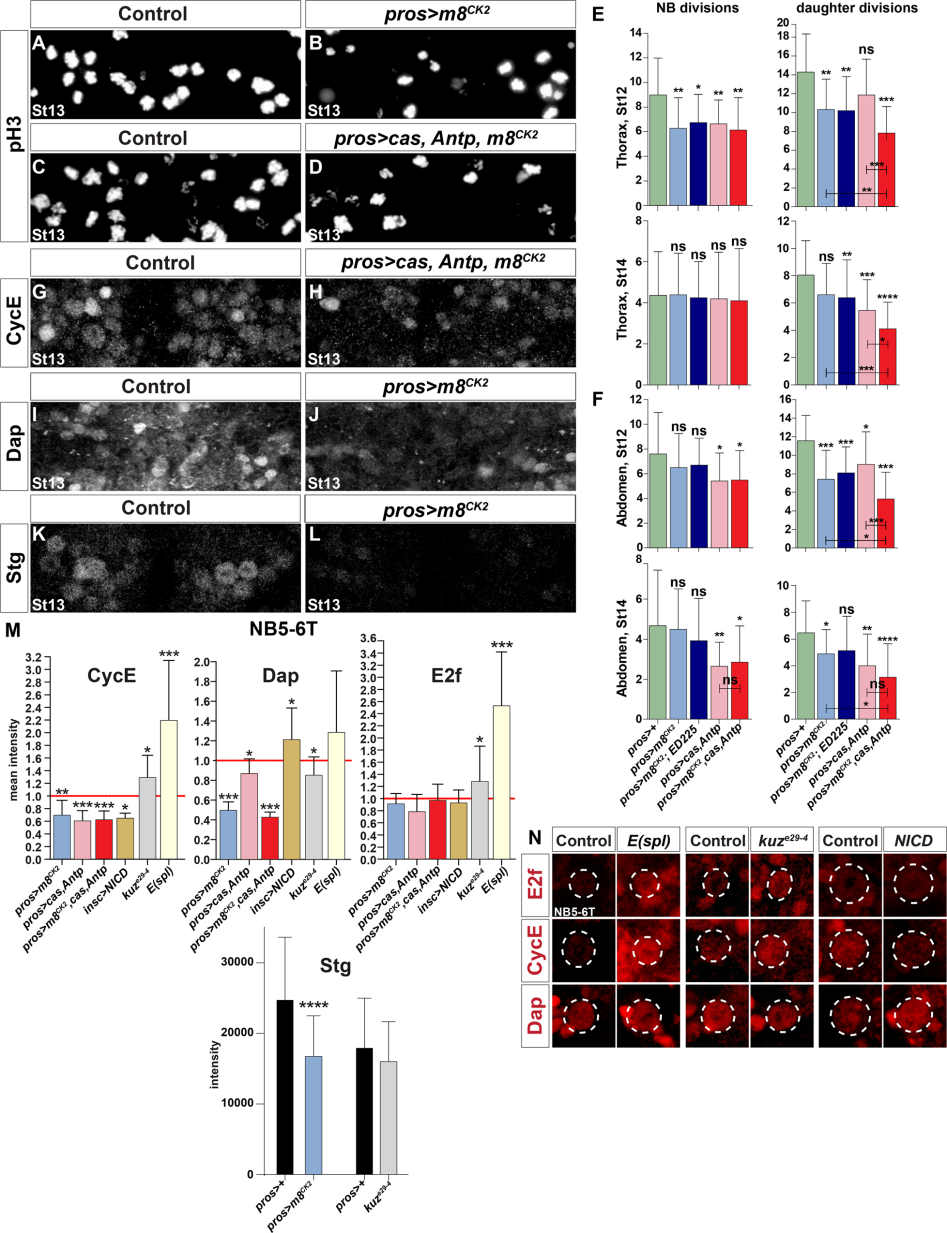
Proliferation and cell cycle regulator effects in Notch pathway perturbations. (A-D) Proliferation (pH3+ cells) is reduced in embryos misexpressing *m8*^*CK2*^ and *m8*^*CK2*^, *Antp*, *cas*, when compared to control (one thoracic segment). (E-F) Quantification of NB and daughter proliferation, in T2-T3 (E) and A1-A2 (F) (* p≤0.05, ** p≤0.01, *** p≤0.001; +/-SD; Student’s two-tailed T-test; n≥18 segments). (G-L) Expression of CycE, Dap and Stg; one thoracic segment; Dpn was used to identify NBs. (G-H) Triple misexpression of *m8*^*CK2*^, *cas* and *Antp* reduces CycE expression. (I-L) Misexpression of *m8*^*CK2*^ reduces Dap and Stg expression. (M-N) Expression of CycE, E2f and Dacapo (Dap) in the NB5-6T neuroblast, at St13-14 (* p≤0.05, ** p≤0.01, *** p≤0.001; +/-SD; Student’s two-tailed T-test; n≥12 NBs).

In contrast to the frequent use of the broad Notch pathway activator NICD, fewer studies have demonstrated effects from misexpressing the various *E(spl)-HLH* genes. We tested a number of available *E(spl)-HLH UAS* transgenes, but observed little if any effects upon the number of Ap cells in NB5-6T. The *E(spl)-HLH* genes are controlled by miRNAs [38], and to circumvent this level of regulation, we generated a novel *UAS* transgene for *m8*; avoiding both the 5´and 3´UTR, and codon-optimizing the open-reading-frame (S1 Data). A FLAG epitope tag was furthermore added to the N-termini (S5A Fig). Surprisingly, these changes to the RNA sequence did not result in any clear expression when driven from *pros-Gal4*, as judged by FLAG epitope antibody stain (S5B and S5C Fig). In addition to miRNA control of *E(spl)-HLH* expression, these genes are however also controlled at the post-translational level e.g., by phospho-degron mediated proteolysis on a Casein Kinase 2 (CK2) site [39–41]. We therefore mutated the CK2 site in m8 (S1 Data; S6A Fig), and observed that the *UAS-m8*^*CK2*^ transgene showed robust FLAG-tag expression in the embryonic CNS (S6D and S6G Fig). Using this transgene, we found that expression of *m8*^*CK2*^ from *pros-Gal4* resulted in reduction of daughter proliferation, evident in both the thorax (St12) and abdomen (St12 and St14) (Fig 5A and 5B and 5E and 5F). We also noted effects on NB proliferation, but only in the thorax at St12 (Fig 5E and 5F). In line with these results, analysis of the NB5-6T lineage revealed a reduction in the total number of cells generated in this lineage in *pros*>*m8*^*CK2*^, from around 17 cells in control to some 13 cells in misexpression (S7A Fig).

We conclude that ectopic *E(spl)-HLH* expression can trigger a premature Type I>0 switch, without strongly affecting NB proliferation, and that triggering a premature switch logically leads to reduction of cell numbers generated in a lineage.

### *E(spl)m8* Can Act Combinatorially with the Temporal Gene *castor* and Hox Gene *Antennapedia* to Trigger Premature Type I>0 Daughter Proliferation Switch

We recently found that the Type I>0 daughter proliferation switch is under control also of the late temporal gene *castor* (*cas*) and the Hox gene *Antennapedia* (*Antp*)[23]. Cas is part of the temporal cascade of transcription factors (Hb>Kr>Pdm>Cas>Grh) playing out in most, if not all, NBs [42]. Antp is gradually expressed in NBs over time, and hence also shows a temporal expression profile [23, 43].

Previous studies did not reveal cross-regulation in NBs between *cas*, *Antp* or Notch signaling [23, 27]. We therefore addressed if *m8* can act combinatorially with *cas* and *Antp*. First, looking at *pros>cas-Antp* co-misexpression, as anticipated from previous studies [23], we noted reduction of daughter proliferation in both thorax and abdomen (Fig 5E and 5F). In contrast to Notch signaling, both *cas* and *Antp* are also involved in the control of NB proliferation exit at the end of lineage development [23]. Indeed, *pros>cas-Antp* co-misexpression resulted in reduction also of NB proliferation, in both thorax and abdomen, at both St12 and St14 (Fig 5E and 5F). Next, we co-misexpressed *m8*^*CK2*^ with *cas-Antp*, and observed striking combinatorial reduction of daughter proliferation, in both thorax and abdomen, at both St12 and St14 (Fig 5C and 5D and 5E and 5F). Similar to *cas-Antp* co-misexpression, we also noted reduced NB proliferation in *m8*^*CK2*^-*cas-Antp* co-misexpression, but this was not significantly increased from that observed in *cas-Antp* co-misexpression (Fig 5E and 5F).

We conclude that stabilized *m8* can act strongly combinatorially with *cas* and *Antp* to trigger a premature Type I>0 switch. In addition, misexpression of all three genes can to a lower degree reduce NB proliferation, but does not act combinatorially in this regard.

### Notch Signaling Affects Expression of Key Cell Cycle Proteins

To further address the connection between Notch signaling and the cell cycle, we analyzed the expression of the key cell cycle proteins described above. Focusing first on NB5-6T, we found upregulation of CycE and E2f in *kuz*^*e29-4*^ mutants, while Dap was down-regulated (Fig 5M and 5N). In the *Df(3R)gro32*.*2*, *P-gro* deficiency, which removes all *E(spl)* genes, E2f and CycE were strongly up-regulated, while Dap was unaffected. In *insc>NICD* we found down-regulation of CycE and upregulation of Dap, whereas E2f was unaffected. Both *pros>m8*^*CK2*^ and *pros>cas-Antp* resulted in CycE and Dap down-regulation, while E2f was unaffected. Triple co-misexpression; *pros>m8*^*CK2*^*-cas-Antp*, did not differ from *m8*^*CK2*^ alone or *cas-Antp* co-misexpression (Fig 5M and 5N). In addition to the effects observed after detailed quantification in NB5-6T, several changes were readily observed globally in NBs: down-regulation of CycE in *pros>m8*^*CK2*^*-cas-Antp*, down-regulation of Dap in *pros>m8*^*CK2*^, and Stg down-regulation in *pros>m8*^*CK2*^ (Figs 5G–5L and S7B). Hence, we find changes in protein expression of all four key cell cycle proteins in Notch/E(spl)-HLH mutants and misexpression embryos.

### Genome-Wide DNA-Binding Analysis Reveals Overlapping Targets for Su(H) and E(spl)

Our genetic interaction and protein expression analysis shows that the Notch/E(spl)-HLH pathway regulates four key cell cycle genes. To address the molecular mechanisms underlying this regulation, we performed Chromatin-Immuno-Precipitation (ChIP) on Su(H) and m8^CK2^, as well as DNA adenine methyltransferase identification (DamID) on m8 and m5, both un-driven and driven by Gal4. Duplicates were conducted for all experimental set-ups, apart from ChIP of m8, which was only conducted once, with similar results.

**Fig 6:**
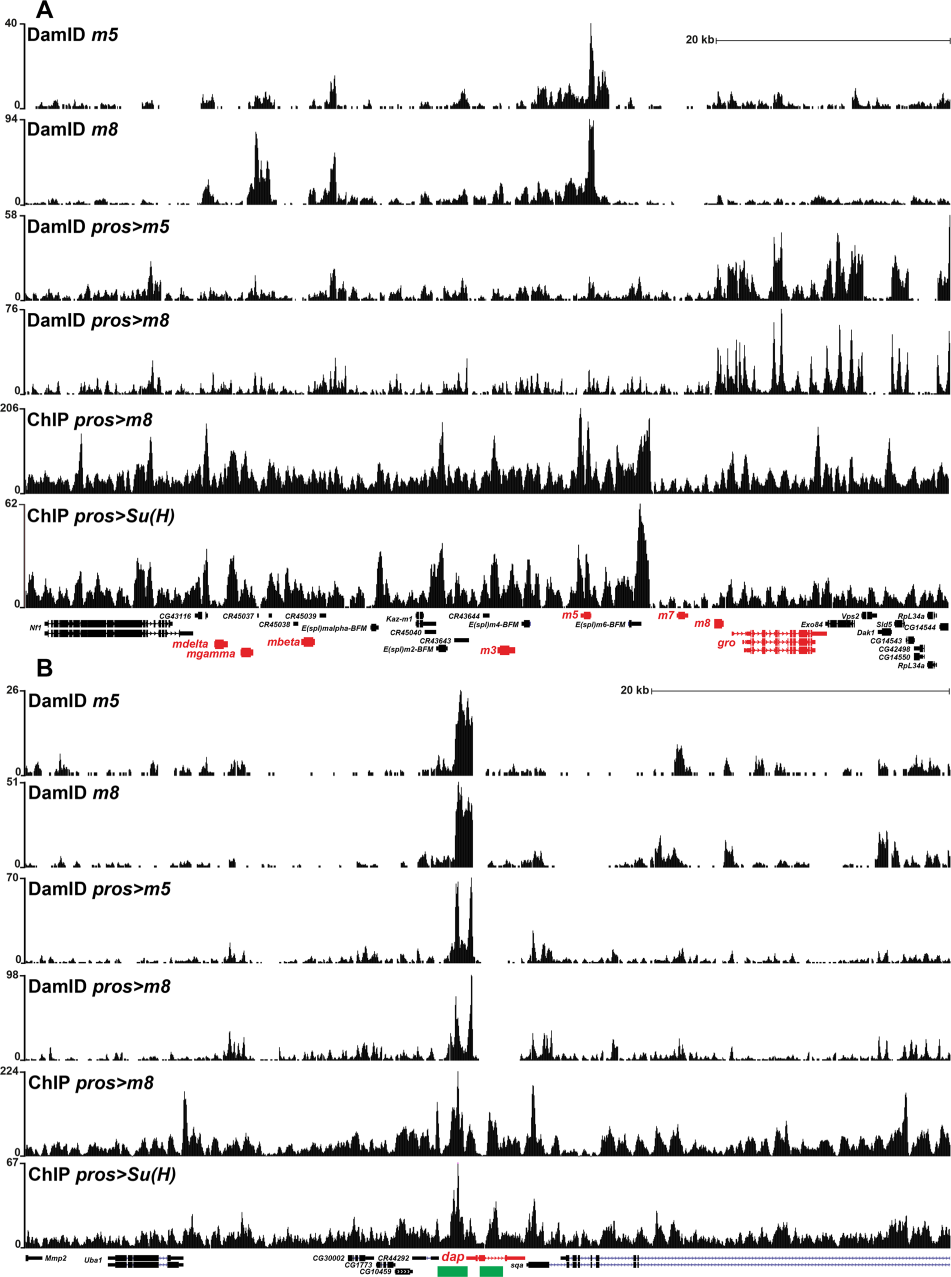
ChIP and DamID analysis of Su(H), m5 and m8 reveals binding to the *E(spl)* complex and *dacapo*. (A-B) Normalized binding profiles for ChIP of FLAG-tagged Su(H) and m8^CK2^, driven by *pros-Gal4*, as well as DamID, driven by *pros-Gal4* or “un-driven”, for m5 and m8. Depicted is the *dap* gene and the *E(spl)* complex. (A) A complex prolife of peaks were detected on the *E(spl)* complex. (B) Two major peaks were detected on *dap* in all six conditions tested, and for all three proteins. These two peaks correspond to previously identified *dap* CNS enhancers (green) [66, 67].

Previous studies of Su(H) have involved ChIP analysis in cell lines and wing disc cells, and have identified binding to the *E(spl)* complex, as well as the *CycE* and *stg* cell cycle genes [4, 12, 44, 45]. To direct our analysis specifically to the developing CNS, we expressed a FLAG-tagged Su(H) construct (S1 Data), driven by *pros-Gal4*, and used the FLAG tag to immunoprecipitate Su(H). In both replicates we identified peaks at the *E(spl)* complex (Fig 6A). Focusing on the key cell cycle genes, we also identified peaks at *CycE*, *dap* and *stg* (Figs 6B and S8A and S8B).

The E(spl)-HLH proteins have not been previously analyzed with respect to chromatin interactions, presumably due to their instability. We performed ChIP on *pros>m8-FLAG* embryos but, not surprisingly, were unable to obtain sufficient amounts of DNA for sequencing. Hence, we turned to *pros> m8*^*CK2*^*-FLAG* embryos, and were now able to obtain sufficient amounts of DNA for sequencing. As has been predicted from previous genetics, m8 binds to the *E(spl)* complex (Fig 6A). In addition, we find peaks on *CycE*, *dap* and *stg* (Figs 6B and S8A and S8B).

To complement the ChIP analysis we turned to DamID, and because of the sensitivity of this assay, performed this technique on embryos only carrying *UAS-m8-DamID* or *UAS-m5-DamID*, allowing for the leakiness of the *UAS* transgene to provide low expression levels [46]. However, given the low stability of the m5 and m8 proteins, we also performed DamID on embryos where each *UAS* was driven by *pros-Gal4*. These experiments also revealed binding of both m8 and m5 to the *E(spl)* complex, as well as to *CycE*, *dap* and *stg* (Figs 6A and 6B and S6A and S6B). Strikingly, Su(H), m5 and m8 peaks overlap with known CNS enhancers, particularly for *dap*, and to some extent for *CycE* and *stg* (Figs 6B and S8A and S8B).

## Discussion

**Fig 7:**
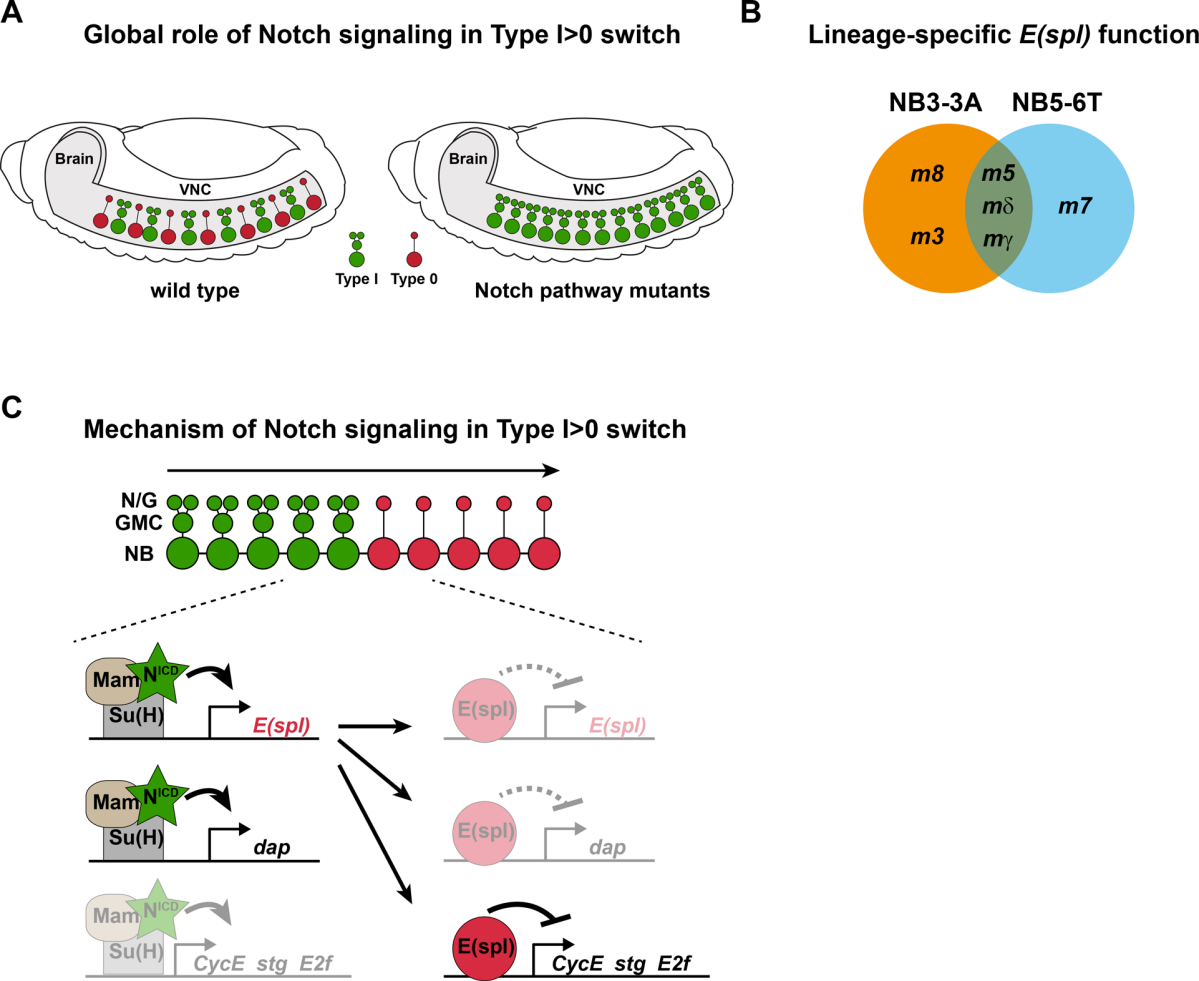
Notch signaling controls the global Type I>0 switch via cell cycle gene control. (A) During *Drosophila* VNC development many, perhaps all, NBs undergo a programmed Type I>0 daughter proliferation switch, mediated by the Notch pathway. (B) Analysis of two distinct lineages, NB3-3A and NB5-6T, using novel TILLING, recombineering and CRISPR/Cas9 alleles, reveals the differential function of *E(spl)-HLH* genes in the Type I>0 switch. (C) Genetic and molecular studies support a multi-levelmodel, where Notch signaling (NICD-Mam-Su(H)) initially activates *E(spl)-HLH* and *dap*, and where E(spl)-HLH subsequently represses *CycE*, *stg* and *E2f*. We also find evidence for partial regulation of *CycE*, *stg* and *E2f* by NICD-Mam-Su(H), and of *dap* and *E(spl)-HLH* by E(spl)-HLH (shaded; see text for details).

Most if not all NBs commence lineage progression by dividing in the Type I mode, but subsequently many switch to Type 0 mode [23, 27, 47]. We find that Notch signaling acts globally in the VNC to trigger this Type I>0 switch, and that critical downstream genes are the *E(spl)* genes (Fig 7A and 7B). In addition, our recent survey of 21 *Drosophila* cell cycle genes, combined with an extensive genetic screen, identified critical input from the *CycE*, *stg*, *E2f* and *dap* genes [23, 36]. Here, we find evidence for direct links between the Notch pathway, *E(spl)-HLH* and these four cell cycle genes. Several genetic and molecular findings help to resolve these connections: First, we observe genetic interaction between Notch components (*kuz* and *E(spl)-C*) and cell cycle genes (*CycE* and *dap*; Figs 4 and S5). Second, *kuz* and *E(spl)-HLH* mutants show elevated daughter proliferation, while expression of NICD and m8^CK2^ shows reduced daughter proliferation. Third, with respect to cell cycle protein expression, both the *kuz* and *E(spl)-HLH* mutants, as well as NICD and m8^CK2^ expression, affects both cell cycle activators (CycE, Ef2, Stg) and inhibitors (Dap) (Fig 5). Finally, the ChIP and DamID results suggest that most, if not all of these interactions involve direct transcriptional regulation. Based on these findings, we propose a multi-level model, where Notch signaling (NICD-Mam-Su(H)) first directly activates *E(spl)-HLH* and *dap*, and where *E(spl)-HLH* subsequently directly represses itself, *CycE*, *E2f*, *stg* and *dap* (Fig 7C).

However, conflicting with this model is the finding that while Su(H) binds to *CycE*, NICD expression in fact triggers down-regulation of CycE. The explanation for this dichotomy may be that while the NICD-Mam-Su(H) complex presumably activates CycE, the simultaneous activation of E(spl)-HLH proteins, which also bind *CycE* and are obligate repressors, would nevertheless result in repression of CycE. Another conflicting result is that while m8^CK2^ represses CycE, Stg and Dap expression, it still reduces daughter proliferation. Presumably, the down-regulation of Dap, which should trigger daughter proliferation [23], is counteracted by the reduction of both CycE and Stg. This somewhat conflicting evidence for both first and second level regulation of both cell cycle drivers and inhibitors is presumably necessary to avoid a complete stop of lineage progression. Specifically, if NICD-Mam-Su(H) exclusively activated Dap and E(spl)-HLH, and E(spl)-HLH in turn exclusively repressed CycE, Stg and E2f, then proliferation would presumably stop completely even at very low levels of Notch activation. Hence, the balanced counterplay between activator and repressor Notch output, upon both cell cycle drivers and inhibitors, allows for a precise daughter proliferation switch, while allowing for lineage progression to continue. This dichotomous nature of the Notch pathway–regulating both activators and suppressors in a pathway–has been previously observed, and was coined “incoherent network logic” [4], and may well be a very common feature of Notch signaling.

### Combinatorial Control of the Type I>0 Daughter Proliferation Switch

The Type I proliferation mode is to great extent controlled by the Pros homeodomain transcription factor. Pros is expressed by most, if not all NBs, is cytoplasmically located in NBs, and distributes asymmetrically to the daughter cell where it enters the nucleus [48–50]. Once in the daughter nucleus, Pros acts to repress key cell cycle genes i.e., *CycE*, *stg* and *E2f* [46, 51], thus ensuring that the daughter can only divide one time; Type I. In contrast, Pros does not appear to play a central role for the Type 0 proliferation mode [23, 27]. Instead, the Type I>0 switch is controlled by several cues emerging late in NB lineage progression. In the thorax, these include the temporal gene *cas* and the Hox gene *Antp*, both of which are selectively expressed during latter stages of NB lineage development [23, 43]. Additionally, our studies demonstrate that the Notch pathway is also involved in the Type I>0 switch (this study; [27]). Because the Notch pathway is off in early delaminating NBs–a prerequisite for the generation of NBs–but is gradually activated in NBs [27], Notch also acts as a temporally gated cue for the Type I>0 switch. Misexpression of *cas* and *Antp* can trigger a premature Type I>0 switch [23], and here, we find that activation of the Notch pathway (NICD or m8^CK2^) can act similarly. Our previous studies demonstrated that Notch, *cas* and *Antp* do not regulate each other [23, 27]. In line with this finding, we find that *m8-cas-Antp* co-misexpression shows combinatorial effects on the Type I>0 switch.

A fascinating feature of *Drosophila* embryonic NB lineages is that the Type I>0 switch occurs at different and reproducible stages of lineage development in each NB type e.g., a short Type I window in NB3-3A, but a long Type I window in NB5-6T. Our results indicate that temporal, Hox and Notch input, acting in parallel pathways on partly overlapping but also distinct cell cycle genes, combinatorially contribute to high fidelity and lineage-specific flexibility for the timing of the Type I>0 switch. In doing so, they must somehow integrate with earlier pattering mechanisms to ensure the NB-specific timing of the Type I>0 switch.

### DNA-Binding Profiles of Su(H) and E(spl)-HLH Proteins

There is a well-established regulatory connection between Su(H) and the *E(spl)-HLH* genes. This stems from DNA-binding and enhancer studies, demonstrating Su(H) binding to key regulatory elements in the enhancers of several *E(spl)-HLH* genes [7, 9]. In addition, several studies, in cell lines and wing disc cells, have demonstrated binding of Su(H) to the *E(spl)* complex using ChIP [4, 12, 44, 45]. These studies also revealed binding of Su(H) to *CycE* and *stg*. In contrast, the direct targets of the E(spl)-HLH proteins are less clear, and only a subset of Notch targets have been identified as direct targets [52]. To our knowledge, there are no genome-wide data for any HES protein, presumably due to the instability of these proteins.

Analyzing our ChIP and DamID results, we find that the target genes *E(spl)-C*, *CycE*, *dap* and *stg* fall into several categories. For the *dap* target gene there is overall agreement between the two methods used and the three different proteins (Su(H), m5 and m8); peaks are overlapping, and fit with two known enhancer elements (Figs 6A and 6B and S6A and S6B). In contrast, for the *CycE* target gene, a more complex picture emerges. First, although DamID for m5 and m8 show very similar profiles, there are striking differences in the peak profiles of driven versus un-driven m5 and m8: un-driven m5 and m8 show one major peak in the *CycE* promoter, whereas driven m5 and m8 show a set of peaks in the intronic region. Second, m8 ChIP only partially overlaps with driven m5 and m8 DamID. Third, ChIP for Su(H) shows a somewhat different profile, with several peaks not matching m5 and m8 DamID and ChIP peaks (Figs 6A and 6B and S6A and S6B). For *stg* there is overall agreement between driven and un-driven m5 and m8, but here the ChIP for m8 stands out, with a set of three very strong peaks in the upstream region. Regarding differences between different DamID experiments, one reason for different profiles when comparing driven versus un-driven may be that un-driven DamID relies upon low-level ubiquitous leakiness of the *UAS* transgene, whereas driven DamID is activated by a CNS-specific *Gal4* driver. Regarding differences between DamID and ChIP, it is generally assumed that DamID detects also transient binding, whereas ChIP relies more upon persistent binding to the DNA. Regardless of these differences in profiles, observed using the different experimental approaches, we believe that the ChIP and DamID results support the notion of direct regulation of key cell cycle genes by NICD-Mam-Su(H) and E(spl)-HLH.

### Specificity of *E(spl)-HLH* Genes

A number of studies have attempted to address the possible specificity of the seven *Drosophila E(spl)-HLH* genes. Loss-of-function studies, using deletions, have resulted in the notion that these seven genes are highly redundant, supported also by the fact that no individual loss-of-function phenotypes have been identified [13–16]. In contrast, gain-of-function studies have lent support for a notion that while different E(spl)-HLH proteins indeed have similar function, they may differ in their efficiency in promoting different developmental outcomes [53].

Here, by using single-lineage analysis, and in a sensitized background–removing half a copy for all *E(spl)-HLH* genes–we find that six out of the seven *E(spl)-HLH* genes are involved in the Type I>0 switch (Fig 7C). The fact that *m3* and *m8* only act in NB3-3A and *m7* in NB5-6T likely reflects differential gene expression–NB specificity and/or levels–rather than differential protein function, since the biological output is the same: the Type I>0 switch. All seven *E(spl)-HLH* genes are known to be expressed in the developing VNC, in a salt-and-pepper fashion [54], and we previously used an *m8-GFP* reporter to reveal that Notch signaling commences in the NB5-6T NB during latter stages of lineage development [27]. This reporter expression was dependent upon Notch signaling, evident by the loss of expression in NB5-6T in *kuz*^*e29-4*^. Here, we also find expression of *m8-GFP* also in NB3-3A, in line with the role of *m8* in this lineage. We have made extensive efforts aimed at generating reporter transgenes for all seven *E(spl)-HLH* genes, and antibodies to their protein products, but this has not resulted in reproducible detection of expression in NBs. Hence the details of the expression of all seven E(spl)-HLH in different NBs remain unclear.

It is tempting to speculate that rather than a high degree of specificity of expression in different NBs, *E(spl)-HLH* genes may act in a generic additive manner. This notion is in part supported by our findings: NB3-3A has an early Type I>0 switch, which involves five *E(spl)-HLH* genes, while NB5-6T shows a later switch, involving four genes. Another intriguing idea is that different *E(spl)-HLH* genes may be utilized for the Type I>0 switch during different time-windows i.e., a temporal *E(spl)-HLH* cascade. However, we find no evidence for this idea in our results, since the different *E(spl)-HLH* mutants show similar numbers of aberrant daughter proliferation at the same stage (Fig 3G–3I and 3N-3P).

### Multi-level Notch Effector Output; a Dynamic System for Proliferation Control

The Notch pathway is controlled at a number of different levels, including miRNA and protein-stability control of most, if not all components [55–57]. Our findings here add further complexity to Notch regulation, by proposing feedforward activation and negative feedback between primary- and secondary-level TFs in the pathway, as well as by both activation and repression of an overlapping set of key cell cycle regulators (Fig 7C). This regulatory model is especially intriguing when viewed against a growing body of evidence that points to the importance of oscillations of Notch signaling with respect to differential biological outcomes [58]. Support for complex interplay between Notch signaling and the cell cycle recently emerged also from mathematical modeling [59]. This regulatory interplay combines for a highly flexible and dynamic signaling output, and suggests that variations in Notch signal strength and length may help explain the anti- or pro-proliferative output from this pathway.

## Materials and Methods

### Immunohistochemistry

A DNA fragment encoding Stg protein was expressed in bacteria. Protein was PAGE-gel purified and injected into guinea pigs, mice and rats. See S1 Text: Extended Experimental Procedures for details.

### Fly Stocks

*E(spl)-HLH* TILLING alleles were obtained by TILLING (Targeted Induced Local Lesions IN Genomes) of all seven *E(spl)-HLH* genes on the Fly-TILL platform [60]. Gene-specific deletion mutants were generated from a functional *E(spl)-C* BAC [61], that was modified in three consecutive steps of recombineering mediated gap-repair [62]. The resulting transgene was integrated at the M[3xP3-RFP, attP]51D attP site using phiC31-mediated integration [62]. CRISPR/Cas9-mediated homologous recombination was used to generate a null allele of *E(spl)-HLH- mγ*. See S1 Text: Extended Experimental Procedures for details.

### UAS Transgenes

Novel UAS transgenes were generated for *Su(H)* and *m8* by de-novo gene synthesis (Genscript, Piscataway, NJ, USA). DNAs were inserted into the pUASattB vector, and transgenes generated by PhiC31 transgenic integration [63] (BestGene Inc. Chinmo, USA). UAS-TF-Dam transgenic flies were generated by *P* element transformation (BestGene Inc. Chinmo, USA). See S1 Text: Extended Experimental Procedures for details.

### DNA Adenine Methyltransferase Identification (DamID)

*Drosophila* DNA adenine methyltransferase identification (DamID) was carried out according to a modified protocol based on a method from Vogel et.al. [64] and A. Brand (www.flychip.org.uk). See S1 Text: Extended Experimental Procedures for details.

### Chromatin Immunoprecipitation (ChIP)

Chromatin preparation was carried out according to the protocol from Négre et al., (http://wiki.modencode.org/project/uploads/6/6b/ChIP_protocol_NN_07v1.2.pdf). Immunoprecipitation was conducted according to MERCK Millipore protocol (Manga ChIP protein A/G beads), using αFLAG (m) 1:200 (BPS Bioscience cat: 25003). See S1 Text: Extended Experimental Procedures for details.

### DNA Sequencing and Bioinformatics

Sequencing was carried out on the Illumnina HiSeq2500 platform. DNAstar Seqman NGN software (DNASTAR, Inc. version 12.2) was used for sequence assembly. Normalization was done with RPM, Qseq was used for peak detection and the wig-files were aligned to genome assembly dm6 on the UCSC genome browser for visualization [65]. See S1 Text: Extended Experimental Procedures for details.

## Supporting Information

S1 Text Extended Experimental Procedures and Supplemental References. (DOCX)

S1 Data DNA sequences of the synthetic Su(H) and E(spl)m8-HLH constructs. (PDF)

S1 Fig (Related to Fig 1) Notch controls the Type I>0 switch in NB5-6T. (A-E) The last-born cells in the NB5-6T lineage, the Ap neurons, are born as Type 0 cells; 4 cells in T2-T3 and 5 cells in T1; identifiable by Eya, and neuropeptides FMRFa and Nplp1. (B) *kuz*^*e29-4*^, *elav>Su(H)RNAi* (C), and (D) *insc>Tom;neur*^*Df*^*/+*, frequently displayed extra Eya cells, as well as Ap1 and Ap4 duplications. (E) Although there is no apoptosis in the latter part of the NB5-6T lineage in wild type [26], previous studies demonstrated that when the Type I>0 switch is perturbed, and daughters undergo aberrant divisions, some ectopic daughters may undergo apoptosis [23]. Therefore, to reveal the full proliferation effect of *kuz* mutants we combined *kuz*^*e29-4*^ with a programmed cell death (PCD) mutant (*Df(3L)ED225*). However, this did not result in increased Ap cell numbers beyond that observed in *kuz*^*e29-4*^ alone. (F) Quantification of Eya cell numbers (* p≤0.05, ** p≤0.01, *** p≤0.001; n≥32 clusters; Wilcoxon signed-rank test; +/-SD). Previously, we determined that the Delta ligand was involved in this Notch event [27]. We analyzed *Ser* mutants and a recently generated *Notch* mutant, *Notch*^*jigsaw*^, which only affects the interaction between Notch and Ser, leaving Notch-Delta interactions un-perturbed [68]. We did however not observe any changes in Ap cell number in either mutant. (G) The NB5-6T lineage progresses with nine Type I rounds of asymmetric divisions followed by five Type 0 divisions [2]. In Notch pathway perturbations, the Type I>0 switch is perturbed leading to aberrant divisions of daughter cells. (M) Cartoons illustrating the proliferation effects in cell death (PCD), *kuz*^*e29-4*^ (Notch) and PCD, *kuz* double mutants (black squiggle depicts dividing cells; Q = quiescence; exit = cell cycle exit; see Fig 2G and 2H for data). Previous studies have revealed that NBs in the VNC can stop lineage progression in three distinct ways: by programmed cell death (PCD) (“stop-by-PCD”; exemplified by NB7-3) [23,69]; by cell cycle exit followed by PCD (“stop-then-PCD”; exemplified by NB5-6T) [26, 43]; and by cell cycle exit followed by quiescence (“stop-then-Q”; exemplified by NB3-3T) [70]. “Stop-by-PCD” may be a rare event–in fact so far it has only been reported in one NB (NB7-3). In line with this notion, while previous studies have revealed that a subset of thoracic NBs and the majority of abdominal NBs are removed by PCD during mid to late embryogenesis [71–73], there has been no report of global over-proliferation of the embryonic VNC in PCD mutants. Our results (Fig 2G and 2H) are in line with previous studies of PCD in NBs and of proliferation control. In wild type, a small fraction of NBs terminate lineage progression by “Stop-by-PCD”, while the majority terminates via either “Stop-then-PCD” (majority of abdominal NBs) or “Stop-then-Quiescence” (the majority of thoracic NBs; Fig 1S). All three categories have likely, to great extent, undergone the Type I>0 switch prior to lineage termination [23]. In PCD mutants, a small increase in NB proliferation is observed, specifically in the “Stop-by-PCD” NBs, while daughter proliferation is generally unaltered. In Notch pathway mutations, NB proliferation is unaffected, while the Type I>0 switch is affected. In PCD-Notch double mutants, the few “Stop-by-PCD” NBs that now survive add very minimally to the Type I>0 switch phenotype. Our findings are hence in line with previous findings, and we find minimal effects on NB proliferation in PCD mutants (*Df(3L)ED225*), with only one of four time-points studied showing a small increase. Similarly, analysis of daughter proliferation in PCD mutants revealed minimal effects, with only one of four measurements showing a small increase.
(PDF)

S2 Fig (Related to Fig 3) Genetic redundancy in the *E(spl)* complex. To begin addressing the role of each member of the *E(spl)* complex with respect to the Type I>0 switch, we first analyzed a number of deficiencies in this region, using the NB5-6T lineage and the four Ap neurons (Type 0) as readout. As anticipated, these studies revealed a complex picture of *E(spl)-HLH* gene involvement. The picture is further complicated by the fact that the gene encoding the E(spl)-HLH transcriptional co-repressor Groucho (Gro) is located adjacent to the *E(spl)* complex. (A) Schematic of the *E(spl)* complex, depicting only the *E(spl)-HLH* genes and *gro*; horizontal bars outline the deficiencies used. (B) Crosses made between different deficiencies. Colored overlap indicates homozygous deleted regions (null). (C) Heterozygous deletion of the entire *E(spl)* region, without removing *gro*, did not reveal any significant phenotype at stage air-filled trachea (AFT)(*Df(3R)BSC751/+)*. Similarly, overlapping deletions removing *E(spl)m7-HLH* (*m7*) and *m8* showed no significant effect (*Df(3R)gro32*.*2*, *P-gro/Df(3R)ED6232*; S2B, S2F). (D) In contrast, heterozygous deletion of the region, while also removing one gene copy of *gro*, resulted in extra Ap cells (*E(spl)-C*^*Δmδ-m6*^*/Df(3R)ED6232*; S2B, S2F). Overlapping deletions removing *m3* and *m5*, while also removing one gene copy of *gro*, gave strong effects (*E(spl)-C*^*Δmδ-m6*^*/Df(3R)Exel6204*; S2B, S2F). Removal of *m3*, *m5*, as well as *m7* and *m8*, while rescuing *gro* function, increased these effects (*Df(3R)gro32*.*2*, *P-gro/Df(3R)Exel6204*; S2B, S2F). Removal of *m3*, *m5*, as well as *mδ*, *mγ* and *mβ* also gave strong effects (*Df(3R)gro32*.*2*, *P-gro/E(spl)-C*^*Δmδ-m6*^; S2B, S2F). Further removal of *gro* in this background did not exacerbate this already strong effect (*Df(3R)BSC751/E(spl)-C*^*Δmδ-m6*^; S2B, S2D-F). (E) Expression of Dpn+ reveals extra delaminated NBs in the NB5-6T lineage, marked by *lbe(K)-GFP*. (F) Quantification of Eya+ cells per thoracic T2/T3 Ap cluster +/- SD, in a number of *E(spl)-HLH* composite deletions, at AFT. Asterisks denote significant difference compared with the heterozygous deficiency (*lbe-GFP*, *Df(3R)BSC751/+*) (* p ≤0.05, ** p≤0.01, *** p≤0.001; +/-SD; Annova, Bonferroni posthoc; n≥12 clusters). Extra Eya cells are generated in the majority of crosses, and the severity mirrors the extent of genetic ablation. (G) Based upon these and previous results [27], we find that perturbation of *E(spl)-HLH* genes increases Ap cell numbers by two routes; weaker mutant combinations only affect the Type I>0 switch, while stronger combinations also result in extra NB5-6T generation.
(PDF)

S3 Fig (Related to Fig 3) Eya cell numbers in Notch/E(spl) mutants. (A) Quantification of Eya cells in NB5-6T. The three TILLING alleles identified for *m5* do not show significant effects when crossed to each other. This is in contrast to the results found when these *m5* alleles are placed over a deletion uncovering all seven *E(spl)-HLH* genes (*Df(3R)BSC751*) (S2 Fig). The lack of effect for *m8*^*V59M*^ in NB5-6T (Fig 4) is not likely due to that it is a hypomorphic allele, because the previously identified *m8*^*1*^ also did not show any effect, even when placed over deficiency (*Df(3R)BSC751*) (* p ≤0.05, ** p≤0.01, *** p≤0.001; Kruskal-Wallis, Dunn’s posthoc; +/-SD; n≥40 segments). (B) Expression of GFP in the *E(spl)HLHm8-GFP* transgenic line is observed in NB3-3A, at St13. NB3-3A was identified by position, expression of Eve (not shown; out of the focal plane shown here), Dpn and Cas (known to be selectively expressed by NB3-3A at St13 [74]).
(PDF)

S4 Fig (Related to Fig 4) Notch does not control the Type I>0 switch via regulation of proneural genes. Previous studies of Notch signaling during the process of lateral inhibition demonstrated that key targets are the genes of the proneural family of bHLH transcription factors; *achaete*, *scute* and *lethal-of-scute* [34, 35]. Addressing the possible role of these genes in the Type I>0 switch is not trivial, first because of their genetic redundancy, and second because of their prominent role in lateral inhibition; in compound mutants many NBs fail to form during early neurogenesis [75, 76]. However, arguing against their role in NBs during the subsequent Type I>0 switch is the fact that they are known to be rapidly down-regulated immediately following NB delamination [77, 78]. (A-F) Expression of Sc, L´Sc and Ac in thoracic segments T1-T2, at StE12. The NB5-6T lineage is visualized by *lbe(K)-GFP* and NBs by Dpn. We stained for all three proneural proteins at St12; the time-point at which the Type I>0 switch is in progress globally. While we could detect all three proteins in a minor subset of neurons and glia, we found little if any expression in wild type NBs (A, C, E). We furthermore analyzed Ac, Sc and L-sc expression in *kuz*^*e29-4*^, but did not observe any apparent activation of these proteins, in NBs or other cells (B, D, F). While we cannot completely rule out involvement of the proneural genes in the Type I>0 switch, their rapid down-regulation in early NBs, their apparent lack of expression in NBs at the time of the switch, and the lack of effects on proneural expression in *kuz* mutants, strongly argues against proneural involvement in this Notch function.
(PDF)

S5 Fig (Related to Fig 5) Activated Notch reduces daughter proliferation. (A) Quantification of the number of NB3-3A cells, at St17 (*eg-Gal4/UAS-GFP*). While *dap* or *kuz* heterozygotes do not show significant increase in NB3-3A lineage cells, *kuz*/*dap* transheterozygotes show clear effects. These effects are similar to those observed in *kuz* or *dap* homozygotes (* p≤0.05, ** p≤0.01, *** p≤0.001; n≥47 lineages; ANOVA with Dunnett’s posthoc test; +/-SD). (B) Quantification of dividing NBs and daughters in the VNC in *insc>NICD* expression versus control; at two different developmental stages; in the thorax and abdomen (T2-T3 and A1-A2) (* p≤0.05, ** p≤0.01, *** p≤0.001; Student’s two-tailed T-test; n≥20 segments; +/-SD). Reduced daughter proliferation is observed at St12, in both thorax and abdomen, while NBs are unaffected.
(PDF)

S6 Fig (Related to Fig 5) Generation of stabilized *UAS-m8* transgenes. (A) New *UAS-m8* constructs were generated, omitting the 5´and 3´UTR, and codon optimizing the ORF for m8. The sequence upstream the start-ATG was altered to match the *Drosophila* consensus and the CK2 phospho-degron was mutated. The transgene was inserted at *28E* on chromosome 2. (B-G) Control and embryos expressing the novel *UAS* constructs, driven from *pros-Gal4*, detected by FLAG antibody stain. (B-D) three thoracic VNC segments; (E-G) one thoracic hemi-segment, showing NB5-6T identified by *lbe(K)-GFP* (St15). While expression of the *m8-FLAG* construct is not readily detected above control background, *m8*^*CK2*^*-FLAG* shows robust staining in the VNC and in NB5-6T.
(PDF)

S7 Fig (Related to Fig 5) Stg expression levels and number of cells in NB5-6T are both reduced by E(spl)HLHm8^CK2^ misexpression. (A) Expression of m8^CK2^, driven by *pros-Gal4*, results in significantly reduced cell numbers in the NB5-6T lineage (* p≤0.05, ** p≤0.01, *** p≤0.001; +/-SD; Student’s two-tailed T-test; n≥ 24 lineages). (B) Expression of m8^CK2^, driven by *pros-Gal4*, results in reduced levels of Stg expression. In *kuz*^*e29-4*^ we did not observe significant changes in Stg (* p≤0.05, ** p≤0.01, *** p≤0.001; +/-SD; Student’s two-tailed T-test; n≥26 NBs).
(PDF)

S8 Fig (Related to Fig 6) ChIP and DamID analysis of Su(H), m5 and m8 reveals binding to *Cyclin E* and *string*. (A-B) Normalized binding profiles for ChIP of FLAG-tagged Su(H) and m8^CK2^, driven by *pros-Gal4*, as well as DamID, driven by *pros-Gal4* or “un-driven”, for m5 and m8. Depicted are the *CycE* and *stg* genes. (A) Several peaks were identified on the *CycE* gene, and the profiles differ between the conditions used, most notably between DamID driven by *pros-Gal4* or un-driven. One peak corresponds to a previously identified *CycE* CNS enhancer (green) [79, 80]. (B) On the *stg* gene, a number of peaks were detected in the upstream region. Notable difference is between m8^CK2^ ChIP versus m8 and m5 DamID profiles. Many peaks correspond to previously identified *stg* CNS enhancers (green) [81, 82].
(PDF)

S1 Table (Related to Fig 3) *E(spl)* TILLING mutations. Primer pairs and PCR fragment size used for the TILLING of the seven *E(spl)-HLH* genes. The mutated nucleotide and corresponding amino acid mutation is also shown, as well as the stock numbers from the Stowers collection and the Bloomington stock numbers.
(PDF)
