# Supplementary material for: Control of Neural Daughter Cell Proliferation by Multi-level Notch/Su(H)/E(spl)-HLH Signaling
Source: PLoS Genet. 2016 Apr 12;12(4):e1005984. doi: 10.1371/journal.pgen.1005984 (PMC4829154; doi:10.1371/journal.pgen.1005984)
Supplement: S1 Text — (DOCX) [file pgen.1005984.s001.docx]

**SUPPORTING INFORMATION**

**S1 TEXT 1: EXTENDED EXPERIMENTAL PROCEDURES**

**Immunohistochemistry**

Immunohistochemistry was performed as previously described [[1](#_ENREF_1)]. Primary antibodies were: Guinea pig α-Deadpan (1:1,000) (provided by J.B. Skeath). Rabbit α-phospho-histone H3-Ser10 (pH3) (1:250) (Upstate/Millepore, Billerica, MA, US). Rabbit α-cleaved Caspase-3 (1:100; Cell Signaling Technology, Danvers, MA, US). Rabbit α-GFP (1:1,000) Chicken α-GFP (1:1,000)(Molecular Probes, Eugene, OR, US). Guinea pig α-Dimm (1:1,000), chicken α-proNplp1 (1:1,000) and rabbit α-proFMRFa (1:1,000) [[2](#_ENREF_2)]. Rat mAb α-GsbN (1:10) [[3](#_ENREF_3)] (provided by R. Holmgren). Mouse mAb α-Pros MR1A (1:10), mAb α-Eya 10H6 (1:250) (Developmental Studies Hybridoma Bank, Iowa City, IA, US). Rabbit α-CycE (1:500)(Santa Cruz Biotechnology, Santa Cruz, CA, USA). Guinea pig α-Dap (1:1,000), rat α-E2f (1:100) [[4](#_ENREF_4)].

For the generation of antibodies to Stg, a DNA fragment encoding the full-length Stg protein (*stg* is predicted to only generate one protein variant; http://flybase.org) was generated by codon optimization (for *E.coli* expression) of the open-reading-frame, synthesized by de-novo gene synthesis (Genscript, Piscataway, NJ, USA), inserted into pGEX-4T3 and expressed in bacteria. PAGE-gel purified Stg-Gst fusion protein was injected into 4 guinea pigs, 4 mice (Davids Biotechnologie, Regensburg, Germany) and 4 rats (Agrisera, Umea, Sweden). Sera from two rats gave specific staining of the embryonic VNC, most strongly observed in mitotic NBs. Sera were tested, at 1:500, for specificity by the absence of specific staining in *stg* mutants (*stg^Df(3R)6212^*) and for ectopic Stg expression in *UAS-stg*/*pros-Gal4* (not shown).

**Fly Stocks**

*lbe(K)-EGFP* [[5](#_ENREF_5)]. *lbe(K)-lacZ [*[*4*](#_ENREF_4)*]*. *elav-Gal4* [[6](#_ENREF_6)]. *Df(3R)BSC751/E(spl)-C^Δmδ-m6^*) [[7](#_ENREF_7)]. *prospero-Gal4* on chr 3 (F. Matsuzaki, Kobe, Japan). *m8^1^* (*E(spl)m8-HLH^1^*); *mam^GA345^*; *Su(H)^IB115^*; *insc-Gal4*; *Ser^Df^* = *Df(3R)Exel6208*; *kuz^e29-4^*; *neur^Df^* = *Df(3R)ED5296*; *2xUAS-Tom*; [*Df(3R)BSC751*](http://flybase.org/reports/FBab0045817.html); *Df(3R)Exel6204*; *Df(3R)ED6232*; *Df(3R)Espl22 (groBx22)*; *Df(3R)gro32.2, P-gro* (from Bloomington Drosophila Stock Center). *E(spl)-C^Δmδ-m6^* [[7](#_ENREF_7)]. RNAi of *Su(H)* was performed by crossing previously generated *UAS-shmiR-Su(H)-2056* lines [[5](#_ENREF_5)] to *elav-Gal4*. Mutants were maintained over *GFP*- or *YFP*-marked balancer chromosomes. As control, *OregonR* or *w^1118^* was used. Staging of embryos was performed according to Campos-Ortega and Hartenstein [[8](#_ENREF_8)].

**TILLING Mutants**

*E(spl)* TILLING alleles were obtained by TILLING (Targeted Induced Local Lesions IN Genomes) of all seven E(spl) genes on the Fly-TILL platform [[9](#_ENREF_9)]. Fly-TILL was performed on a large collection of EMS mutagenized stocks, generated in collaboration between Scott R. Hawley, at the Stowers Institute for Medical Research (Kansas City, MO, USA) and Steven Henikoff, at the Fred Hutchinson Cancer Research Center (Seattle, WA, USA). A total of 66 mutations in the seven *E(spl)* genes was identified in the collection, and a subset of mutant stocks predicted to affect gene function were obtained from S. Hawley. All mutations identified by Fly-TILL and used to study *E(spl)* gene function were verified by PCR-amplification and sequencing (data not shown). The 15 *E(spl)* TILLING alleles used in this study have been deposited at Bloomington Drosophila Stock Center. Primers and identified mutations, as well as Stowers and Bloomington stock numbers are listed in Supplemental Table S1.

**Recombineering Mutants**

The *E(spl)-mδ(FRT)-mβ(FRT)-m3^GFP^* transgene contains the entire genomic region of the *E(spl)-C* carrying a mutant version of the *E(spl)m3-HLH* gene, a wild-type version of the *E(spl)mδ-HLH* flanked by two FRT sites and a wild-type version of the *E(spl)mβ-HLH* flanked by two FRT sites (Figure 3B). To avoid disrupting conserved *cis*-regulatory elements, the FRT sites flanking the *E(spl)mδ-HLH* and *E(spl)mβ-HLH* genes were inserted within non-conserved regions. This transgene was generated by modifying the attB-P[acman] BAC encoding the *E(spl)-C* (Chanet et al. Genetics, 2009) in three consecutive steps of recombineering mediated gap-repair (Venken et al. Science, 2006). First, the ORF of the *E(spl)m3-HLH* gene was replaced by sfGFP using the following primers: m3-sfGfp-F: CAA TCC TTA AAA TAC ACA ACA TAA AAC AAC CCA ATC GAT Cat ggt gtc caa ggg cga gga: m3-sfGfp-R: TAA TAT TAG GGA TAA GAT CCA TAT TGG GCA CGC CCA ATT Agg atc cct tgt aca gct cat. In a second step, one FRT site was inserted 213 nt upstream of the *E(spl)mδ-HLH* start codon and another one 284 nt downstream of the *E(spl)mδ-HLH* stop codon using the following primers: mδ-FRT1.2-F: GAT GCT TCG TTG TGA TCG TCG GCG TGT AGG GAA GTT CCT ATT CTC TAG AAA GTA TAG GAA CTT Cag cgt tgt gat cgc agt gcg tgg tgg c: mδ-FRT1.2-R: TAA TTT TAA AAC ATA CAC AAA AAT TTG TTC GAA GTT CCT ATA CTT TCT AGA GAA TAG GAA CTT Ctt aat gag gct aag tgg aag ctc gg. In a third step, one FRT site was inserted 564 nt upstream of the *E(spl)mβ-HLH* start codon and another one 128 nt downstream of the *E(spl)mβ-HLH* stop codon using the following primers to amplify the FRT sites for the second exchange: mβ-FRT1.2-F: CTC TGC TGG GCT GCT GGG AAA GCT ATT CCT GAA GTT CCT ATT CTC TAG AAA GTA TAG GAA CTT Cgg gca gca att gat gat ctc caa gg: mβ-FRT1.2-R: gtc gca cag acc tct taa agg cgg ccc gTC GAA GTT CCT ATA CTT TCT AGA GAA TAG GAA CTT CCA TCC GTC CGT CCG CTC AAG AAA GAC GG. All recombineering events were verified by sequencing the recombined regions. The resulting transgene was integrated at the M[3xP3-RFP, attP]51D attP site using phiC31-mediated integration (Venken et al. Science 2006). Injection was performed by BestGene Inc. (Chinmo, USA).

We next used FLP-FRT mediated recombination in the germline of *tub85D-flp* males to delete from this transgene the *E(spl)mδ-HLH* gene and/or the *E(spl)mβ-HLH* gene, as well as to generate a small and precise *E(spl)mδ-mβ* deletion. All possible recombination events were recovered within the progeny of a single tube. These deletions were characterized by PCR (primers available upon request).

**Crispr/Cas9 Mutants**

To generate a null allele of *E(spl)-HLH- mγ*, we used CRISPR-mediated homologous recombination (HR) to replace its ORF by those of sfGFP. CRIPSR-mediated HR was performed by co-injecting two plasmids encoding gRNAs located 5’ and 3’ relative to the E(spl)-HLH-mγ gene together with a repair plasmid. The following primers were used for U6g-RNA cloning (see <http://flycrispr.molbio.wisc.edu/protocols> for details): gRNA-5’: cttcGTTGTGTGTTGCTAGACCTT; aaacAAGGTCTAGCAACACACAAC. gRNA-3’: cttcGTAACTATTCGATCGCCCCA; aaacTGGGGCGATCGAATAGTTAC. The repair cassette was generated by BAC recombineering in *E. coli* starting from the attB-P[acman]-Ap BAC encoding the *E(spl)-C* described previously [[10](#_ENREF_10)]. Recombineering was performed as described previously [[11](#_ENREF_11)]. The following modifications were introduced:

• two 2-nt deletions in the PAM sequences targeted by gRNA-5’ and gRNA-3’ to prevent cleavage of the repair construct by the gRNAs

• the replacement of the *E(spl)-HLH-*mγ ORF by those of the *sfGFP* gene

• the insertion of a 3xP3-RFP selection marker flanked by loxP sites 3’ to the modified *E(spl)-HLH-* mγ ^null^ gene.

These modifications were introduced using the following primers:

loxPrpsl-F: AAA CGC AAT AGC ACC TCT CTC TGT CCC CTG TAA CTA TTC GAT CGC CCC ACg gcc tgg tga tga tgg cgg g: loxPneo-R: GCC TTG GCA TTG TTT TCG CTT TCT TTT GTC CGA CGA TCG TGT ATG TGC ATt cag aag aac tcg tca aga agg: loxP-F: CTG TCC CCT GTA ACT ATT CGA TCG CCC CAC ata act tcg tat aat gta tgc tat acg aag tta tgt cga cga att cgc gg: loxP-R: TTC TTT TGT CCG ACG ATC GTG TAT GTG CAT ata act tcg tat agc ata cat tat acg aag tta tac tag aga gct tcg ca: rpsl-F: TTG GCC CAT TTT TTG GGG GTT GTT TCT GGC CAG CTA TAT AAG GCC GAT Cag gcc tgg tga tga tgg cgg g: neo-R: TCT CCT TCG TTT TTT TTT TTT TCA GTT GTG GTG TGA AGT AAT CCT ATC Tat cag aag aac tcg tca aga agg: gRNA-5GFP-F: GAA CAA TAA GAA ACA CAC AAA ATG GTG TCC AAG GGC GAG GAG C: sfGfp5'RNA-R: GCT CCT CGC CCT TGG ACA CCA TTT TGT GTG TTT CTT ATT GTT C: GFP-F: TGT TTC TGG CCA GCT ATA TAA GGC CGA TCA TGG AAG GTC TAG CAA CAC ACA ACG: GFP-R: TTC AGT TGT GGT GTG AAG TAA TCC TAT CTA GGA TCC CTT GTA CAG CTC ATC C.

We then used recombineering to fetch a 6.4 kb long DNA fragment from the modified BAC into the multi-copy plasmid pCR2.1 for injection. This generated a repair construct carrying 1.5 kb long 5’ and 3’ homology arms. The following primers were used for this last recombineering step: pCRmγ-F: GGT TTG GAA CGT GGT TCT CAC ACG ATC GAC ACA CCT GCC CTT TTT TGT ATt tgg tta aaa aat gag ctg a: pCRmγ-R: GAG TAG TAA AGC TAT CAA TGT TAA TTG GTT GCG TAT ATA TGG AAT GCC CTc ttc cgc ttc ctc gct cac t. All constructs were sequenced following each recombineering step.

To generate the *E(spl)-HLH-*mγ^null^ allele, 250 embryos from a *y^1^ M{vas-Cas9.RFP-}ZH-2A w^1118^/FM7a, P{Tb^1^}* (BL-55821) were injected with 50 ng/μl of each gRNA plasmid and 250 ng/μl of the repair construct. Six independent RFP-positive lines were obtained. 4/6 lines were tested by PCR for the presence of GFP and for proper HR at the locus. 4/4 lines were correct. One line was kept for further analysis.

**UAS Transgenes**

Novel UAS transgenes were generated for *Su(H)* and *m8*. These were generated by codon optimization of the open-reading-frame (http://www.jcat.de/), synthesized by de-novo gene synthesis (Genscript, Piscataway, NJ, USA), avoiding the 5’ and 3’ UTR (see Supplemental Information 2 for DNA and protein sequences). Mutations in putative or identified phosphorylation epitopes were also introduced, as well as FLAG and/or HA epitope tags (Figure S6A). DNAs were inserted into the pUASattB vector, and transgenes generated by PhiC31 transgenic integration [[12](#_ENREF_12)] into the 28E chromosomal location (BL#9723)(BestGene Inc, CA, USA).

Since Dam expression was toxic in embryos [[13](#_ENREF_13)], we generated UAS-TF-Dam transgenic flies in which the leaky expression of the TF-Dam was blocked using a transcriptional stop cassette (FRTstopFRT). pUASt-FRTstopFRT-DamTF transgenes were obtained by inserting a BamHI-XbaI PCR fragment encoding the TF ORF into the BglI-XbaI site of the pUAStN-Dam vector [[13](#_ENREF_13)] and then a PCR fragment encoding an FRT-stop-FRT cassette (amplified from pGEM4-FRTpolyA, gift from R. Holmgreen; primer sequences available upon request). Plasmids were verified by sequencing prior to P element transformation performed by BestGene Inc. (Chinmo, USA). Following transformation, the FRTstopFRT cassette was removed using FLP-mediated excision. Using this strategy, we generated *UAS-DamE(spl)m5* and *–m8* lines.

**DNA Adenine Methyltransferase Identification (DamID)**

*Drosophila* DNA adenine methyltransferase identification **(**DamID) was carried out according to a modified protocol based on a method from Vogel et.al. [[14](#_ENREF_14)], with changes made by the A. Brand lab ([www.flychip.org.uk](http://www.flychip.org.uk)). In brief, fly crosses were kept on cage for 15h in 26°C overnight. Embryos at stage ~9-16, were collected and dechorinated for 3 min in 4% bleach. Aliquots of 30µl embryos were washed in PBS and stored at -80˚C. For each preparation embryos were homogenized on ice in a mixture of PBS and RNase A and extracted according to the manufacturer protocol (Qiagen Blood and tissue DNA extraction kit). The DNA were eluted in 100µl 60°C MilliQ water. 2.5µg DNA were digested by Dpn1 (Fermentas) for 1h in 37°C in a total reaction volume of 50µl. The digested gDNAs were then purified using Qiagen PCR purification kit. dsAdR adaptor oligonucleotides were ligated to blunt-ended Dpn1 fragments in Fermentas rapid ligation buffer. DpnII digestion were made using DpnII/Mbol (Fermentas) in Buffer R. The digested DNAs were PCR amplified using Clontech advantage cDNA PCR kit in a total reaction volume of 50µl, 21 times elongation. Following PCR the amplified DNAs were purified once more using the Qiagen kit.

**Chromatin Preparation**

*Drosophila* chromatin preparation was carried out according to the protocol from Négre et al. (<http://wiki.modencode.org/project/uploads/6/6b/ChIP_protocol_NN_07v1.2.pdf>). In brief, *pros-Gal4* (*w1118; P{GMR42G10-GAL4}attP2*; Bloomington stock #50168) males were crossed to *UAS* virgins and kept on cage for 15h at 26°C. Embryos at stage ~12-15, were collected and dechorinated for 3 min in 4% bleach. 200-300mg embryos were used for formaldehyde-crosslinking of chromatin. Embryos were homogenized in a potter homogenizer and cross-linked in a 1.8% formaldehyde buffer and incubated for 15min. Cross-linked lysed material was sonicated twice for 15min, 30sec on, 30 sec off, high level (Diagenode Bioruptor). Aliquots of 200µl chromatin were stored at -80°C. From each chromatin preparation a 20µl aliquot was removed and reverse cross-linked to extract DNA. This step was included to calculate DNA concentration in each chromatin aliquot and to test for fragment size after sonication.

**Chromatin Immunoprecipitation (ChIP)**

The protein of interest was immunoprecipitated according to MERCK Millipore protocol (Manga ChIP protein A/G beads). In brief, aliquots of 200µl chromatin were diluted in ChIP dilution buffer and mixed with αFLAG(m) 1:200 (BPS Bioscience cat: 25003) and 20µl magnetic ChIP beads per reaction (Manga ChIP protein A/G beads, MERCK Millipore) and put in a rotator at 4°C overnight. Bead-bound immuno-complexes were washed subsequently in low salt, high salt, LiCl and TE immuno-complex wash-buffer. The ChIP DNA was eluted with ChIP elution buffer at 65°C and 1300 rounds/min for 6h. Immuno-precipitated DNA was extracted by phenol/chloroform/IAA treatment and EtOH precipitation. Final DNA concentration was measured with Qubit (Invitrogen 2.0).

**DNA Sequencing and Bioinformatics**

Sequencing was carried out by GENEWIZ on the Illumnina HiSeq2500 platform, rapid run mode, in a 50bp (ChIP) or 100 bp (DamID) single-read configuration; ~120M reads/lane were obtained. DNAstar Seqman NGN software (DNASTAR, Inc. version 12.2) was used for sequence assembly. Normalization was done with RPM, Qseq was used for peak detection and the wig-files were aligned to genome assembly dm6 on the UCSC genome browser for visualization [[15](#_ENREF_15)].

### Confocal Imaging and Data Acquisition

Zeiss LSM 700 Confocal microscopes were used for fluorescent images; confocal stacks were merged using Zeiss Image browser LSM software (v4.2) or Adobe Photoshop (CS6). Statistic calculations were performed in Graphpad prism software (v6.05). Images and graphs were compiled in Adobe Illustrator (CS6).

### Statistical Analysis

All statistical calculations were performed using GraphPad Prism software (v6.05). To address statistical significance between only two groups, Student’s t-test was used for normal distributed data, and in case of non-Gaussian distribution, nonparametric Mann-Whitney U test was used. Comparing more than two groups, ANOVA and Dunnett’s posthoc test was used for normal distribution, and Kruskal Wallis with Dunn’s posthoc or Wilcoxon signed rank test for non-Gaussian distribution. To evaluate cell cycle protein levels CNS’s from mutants/overexpression and control embryos were dissected in at least three different separated experiments. The staining intensity was measured in mitotic NBs (pH3+) by means of Adobe Photoshop (CS6) and multiplied with the estimated cell volume to compensate for size. The intensity values from each experiment were standardized, and Student´s t-test was performed on the whole dataset to quantify differences.

**SUPPLEMENTAL REFERENCES**

1. Baumgardt M, Karlsson D, Terriente J, Diaz-Benjumea FJ, Thor S. Neuronal subtype specification within a lineage by opposing temporal feed-forward loops. Cell. 2009;139(5):969-82. PubMed PMID: 19945380.

2. Baumgardt M, Miguel-Aliaga I, Karlsson D, Ekman H, Thor S. Specification of Neuronal Identities by Feedforward Combinatorial Coding. PLoS biology. 2007;5(2):295-308.

3. Buenzow DE, Holmgren R. Expression of the Drosophila gooseberry locus defines a subset of neuroblast lineages in the central nervous system. Developmental biology. 1995;170(2):338-49. PubMed PMID: 7649367.

4. Baumgardt M, Karlsson D, Salmani BY, Bivik C, MacDonald RB, Gunnar E, et al. Global programmed switch in neural daughter cell proliferation mode triggered by a temporal gene cascade. Developmental cell. 2014;30(2):192-208. Epub 2014/07/30. doi: 10.1016/j.devcel.2014.06.021. PubMed PMID: 25073156.

5. Ulvklo C, Macdonald R, Bivik C, Baumgardt M, Karlsson D, Thor S. Control of neuronal cell fate and number by integration of distinct daughter cell proliferation modes with temporal progression. Development (Cambridge, England). 2012;139(4):678-89. PubMed PMID: 22241838.

6. DiAntonio A, Haghighi AP, Portman SL, Lee JD, Amaranto AM, Goodman CS. Ubiquitination-dependent mechanisms regulate synaptic growth and function. Nature. 2001;412(6845):449-52. PubMed PMID: 11473321.

7. Bardin AJ, Perdigoto CN, Southall TD, Brand AH, Schweisguth F. Transcriptional control of stem cell maintenance in the Drosophila intestine. Development (Cambridge, England). 2010;137(5):705-14. Epub 2010/02/12. doi: 10.1242/dev.039404. PubMed PMID: 20147375; PubMed Central PMCID: PMC2827683.

8. Campos-Ortega JA, Hartenstein V. The embryonic development of *Drosophila melanogaster*. New York: Springer-Verlag; 1985.

9. Cooper JL, Till BJ, Henikoff S. Fly-TILL: reverse genetics using a living point mutation resource. Fly. 2008;2(6):300-2. Epub 2008/12/23. PubMed PMID: 19098435.

10. Chanet S, Vodovar N, Mayau V, Schweisguth F. Genome engineering-based analysis of Bearded family genes reveals both functional redundancy and a nonessential function in lateral inhibition in Drosophila. Genetics. 2009;182(4):1101-8. Epub 2009/06/17. doi: 10.1534/genetics.109.105023. PubMed PMID: 19528324; PubMed Central PMCID: PMC2728851.

11. Venken KJ, He Y, Hoskins RA, Bellen HJ. P[acman]: a BAC transgenic platform for targeted insertion of large DNA fragments in D. melanogaster. Science (New York, NY. 2006;314(5806):1747-51. Epub 2006/12/02. doi: 10.1126/science.1134426. PubMed PMID: 17138868.

12. Bischof J, Maeda RK, Hediger M, Karch F, Basler K. An optimized transgenesis system for Drosophila using germ-line-specific phiC31 integrases. Proceedings of the National Academy of Sciences of the United States of America. 2007;104(9):3312-7. Epub 2007/03/16. doi: 10.1073/pnas.0611511104. PubMed PMID: 17360644; PubMed Central PMCID: PMC1805588.

13. Choksi SP, Southall TD, Bossing T, Edoff K, de Wit E, Fischer BE, et al. Prospero acts as a binary switch between self-renewal and differentiation in Drosophila neural stem cells. Developmental cell. 2006;11(6):775-89. PubMed PMID: 17141154.

14. Vogel MJ, Peric-Hupkes D, van Steensel B. Detection of in vivo protein-DNA interactions using DamID in mammalian cells. Nature protocols. 2007;2(6):1467-78. Epub 2007/06/05. doi: 10.1038/nprot.2007.148. PubMed PMID: 17545983.

15. Karolchik D, Barber GP, Casper J, Clawson H, Cline MS, Diekhans M, et al. The UCSC Genome Browser database: 2014 update. Nucleic acids research. 2014;42(Database issue):D764-70. Epub 2013/11/26. doi: 10.1093/nar/gkt1168. PubMed PMID: 24270787; PubMed Central PMCID: PMC3964947.
