# Supplementary material for: Control of Neural Daughter Cell Proliferation by Multi-level Notch/Su(H)/E(spl)-HLH Signaling
Source: PLoS Genet. 2016 Apr 12;12(4):e1005984. doi: 10.1371/journal.pgen.1005984 (PMC4829154; doi:10.1371/journal.pgen.1005984)
Supplement: S1 Data — (PDF) [file pgen.1005984.s002.pdf]

SUPPLEMENTAL DNA SEQUENCES

Sequences of synthetic *m8* and *Su(H) UAS* constructs

DNA sequence is followed by protein sequence for each construct. For DNA; EcoRI and XbaI cloning sites underlined, start-ATG underlined-bold and mutated bases green. For protein; FLAG-tag blue-bold; HA-tag purple, bold; spacer red-bold; mutated "Ck2" amino acids green; mutated "deg" amino acids magenta; and Vp16 domain yellow.

m8-FLAG

gaattcaaccaaa**ATG**GCCGACTACAAGGACGACGACGACAAGGGCCCCCCCGGCATGGAGTACA
CCACCAAGACCCAGATCTACCAGAAGGTGAAGAAGCCCATGCTGGAGCGCCAGCGCCGCG
CCCGCATGAACAAGTGCCTGGACAACCTGAAGACCCTGGTGGCCGAGCTGCGCGGCGACGA
CGGCATCCTGCGCATGGACAAGGCCGAGATGCTGGAGTCCGCCGTGATCTTCATGCGCCAG
CAGAAGACCCCCAAGAAGGTGGCCCAGGAGGAGCAGTCCCTGCCCCTGGACTCCTTCAAGA
ACGGCTACATGAACGCCGTGAACGAGGTGTCCCGCGTGATGGCCTCCACCCCCGGCATGTC
CGTGGACCTGGGCAAGTCCGTGATGACCCACCTGGGCCGCGTGTACAAGAACCTGCAGCAG
TTCCACGAGGCCCAGTCCGCCGCCGACTTCATCCAGAACTCCATGGACTGCTCCTCCATGGA
CAAGGCCCCCCTGTCCCCCGCCTCCTCCGGCTACCACTCCGACTGCGACTCCCCCGCCCCCT
CCCCCCAGCCCATGCAGCAGCCCCTGTGGCGCCCCTGGtgataatagtctaga

MADYKDDDDKGPPGMEYTTKTQIYQKVKKPMLERQRRARMNKCLDNLKTLVAELRG
DDGILRMDKAEMLESAVIFMRQQKTPKKVAQEEQSLPLDSFKNGYMNAVNEVSRVMAS
TPGMSVDLGKSVMTHLGRVYKNLQQFHEAQSAADFIQNSMDCSSMDKAPLSPASSGYH
SDCDSPAPSPQPMQQPLWRPW

m8^{Ck2}-FLAG

gaattcaaccaaa**ATG**GCCGACTACAAGGACGACGACGACAAGGGCCCCCCCGGCATGGAG
TACACCACCAAGACCCAGATCTACCAGAAGGTGAAGAAGCCCATGCTGGAGCGCCA
GCGCCGCGCCCGCATGAACAAGTGCCTGGACAACCTGAAGACCCTGGTGGCCGAGC
TGCGCGGCGACGACGGCATCCTGCGCATGGACAAGGCCGAGATGCTGGAGTCCGCC
GTGATCTTCATGCGCCAGCAGAAGACCCCCAAGAAGGTGGCCCAGGAGGAGCAGTC
CCTGCCCCTGGACTCCTTCAAGAACGGCTACATGAACGCCGTGAACGAGGTGTCCCG
CGTGATGGCCTCCACCCCCGGCATGTCCGTGGACCTGGGCAAGTCCGTGATGACCCA
CCTGGGCCGCGTGTACAAGAACCTGCAGCAGTTCCACGAGGCCCAGTCCGCCGCCGA

CTTCATCCAGAACTCCATGGACTGCTCCTCCATGGACAAGGCCCCCCTGgCCCCCGCC
TCCgCCGGCTACCACgCCGACTGCGACTCCCCGCCCCCTCCCCCCAGCCCATGCAGC
AGCCCCTGTGGCGCCCCTGGtgataatagtctaga

MADYKDDDDKGPPGMEYTTKTQIYQKVKKPMLERQRRARMNKCLDNLKTLVAELRG
DDGILRMDKAEMLESAVIFMRQQKTPKKVAQEEQSLPLDSFKNGYMNAVNEVSRVMAS
TPGMSVDLGKSVMTHLGRVYKNLQQFHEAQSAADFIQNSMDCSSMDKAPLAPASAGY
HADCDSPAPSPQPMQQPLWRPW

Su(H)-FLAG-HA

gaattcaaccaaa**ATG**GCCGACTACAAGGACGACGACGACAAGGGCCCCCCGGCATGAAGTCCT
ACTCCCAGTTCAACCTGAACGCCGCCGCCCCCCCGCCATCGCCTACGAGACCACCGTGGT
GAACCCCAACGGCTCCCCCCTGGACCCCCACCAGCAGCAGCAGCAGCAGTCCCAGGACATG
CCCCACTTCGGCCTGCCCGGCCCCCAGCCCCCCTCCTCCCAGCAGCAGCAGCAGCAGCTGC
AGGTGCACCACCAGCAGCAGCAGCAGCAGCAGCAGCAGCAGCAGCAGCAGCAGCACCAGC
AGCAGATGCAGATGTCCCTGCTGCCCGGCCCCTACCGCCCCCACATCGAGGAGAAGAAGCT
GACCCGCGACGCCATGGAGAAGTACATGCGCGAGCGCAACGACATGGTGATCGTGATCCTG
CACGCCAAGGTGGCCCAGAAGTCCTACGGCAACGAGAAGCGCTTCTTCTGCCCCCCCCCT
GCATCTACCTGTTCGGCTCCGGCTGGCGCCGCCGCTACGAGGAGATGCTGCAGCAGGGCGA
GGGCGAGCAGGGCGCCCAGCTGTGCGCCTTCATCGGCATCGGCTCCTCCGACCAGGACATG
CAGCAGCTGGACCTGAACGGCAAGCAGTACTGCGCCGCCAAGACCCTGTTCATCTCCGACT
CCGACAAGCGCAAGCACTTCATGCTGTCCGTGAAGATGTTCTACGGCAACGGCCACGACAT
CGGCGTGTTCAACTCCAAGCGCATCAAGGTGATCTCCAAGCCCTCCAAGAAGAAGCAGTCC
CTGAAGAACGCCGACCTGTGCATCGCCTCCGGCACCAACGTGGCCCTGTTCAACCGCCTGC
GCTCCCAGACCGTGTCCACCCGCTACCTGCACGTGGAGAACGGCCACTTCCACGCCTCCTCC
ACCCAGTGGGGCGCCTTCACCATCCACCTGCTGGACGACAACGAGTCCGAGTCCGAGGAGT
TCCAGGTGCGCGACGGCTACATCCACTACGGCGCCACCGTGAAGCTGGTGTGCTCCGTGAC
CGGCATGGCCCTGCCCCGCCTGATCATCCGCAAGGTGGACAAGCAGATGGCCCTGCTGGAG
GCCGACGACCCCGTGTCCCAGCTGCACAAGTGCGCCTTCTACATGAAGGACACCGACCGCA
TGTACCTGTGCCTGTCCCAGGAGAAGATCATCCAGTTCCAGGCCACCCCCTGCCCCAAGGA
GCCCAACAAGGAGATGATCAACGACGGCGCCTGCTGGACCATCATCTCCACCGACAAGGCC
GAGTACCAGTTCTACGAGGGCATGGGCCCCGTGGCCTCCCCCGTGACCCCCGTGCCCATCGT
GAACTCCCTGAACCTGAACGGCGGCGGCGACGTGGCCATGCTGGAGCTGTCCGGCGACAAC
TTCACCCCCCACCTGCAGGTGTGGTTCGGCGACGTGGAGGCCGAGACCATGTACCGCTGCA
CCGAGACCCTGCTGTGCGTGGTGCCCGAGATCTCCCAGTTCCGCGGCGAGTGGCTGTGGGT
GCGCCAGCCCACCCAGGTGCCCATCTCCCTGGTGCGCAACGACGGCATCATCTACGCCACC
GGCCTGACCTTCACCTACACCCCCGAGCCCGGCCCCCGCCCCCACTGCAACACCCAGGCCG
AGGACGTGATGCGCGCCCGCCAGAACAACAACAACAACAACATCACCTCCATCTCCAACAA
CAACAACTCCAACAACGCCGGCTCCCCCGCCGCCGGCGGCGGCCTGCAGCAGCAGCAGCAG

CAGCACCAGGCCCTGCCCTCCATCTCCGAGGTGCAGTGGAACTCCCACGGCTCCGGCCTGTC
CGGCCCCCCCGGCTACCCCTACGACGTGCCCGACTACGCCtgataatagtctaga

MADYKDDDDKGPPGMKSYSQFNLNAAAPPAIAYETTVVNPNGSPLDPHQQQQQSQD
MPHFGLPGPQPPSSQQQQQLQVHHQQQQQQQQQQQQQHQQQMQMSLLPGPYRPHI
EEKKLTRDAMEKYMRERNDMVIVILHAKVAQKSYGNEKRFFCPPPCIYLFGSGWRRRY
EEMLQQGEGEQGAQLCAFIGIGSSDQDMQQLDLNGKQYCAAKTLFISDSDKRKHFMLS
VKMFYGNGHDIGVFNSKRIKVISKPSKKKQSLKNADLCIASGTNVALFNRLRSQTVSTRY
LHVENGHFHASSTQWGAFTIHLLDDNESESEEFQVRDGYIHYGATVKLVCSVTGMALPR
LIIRKVDKQMALLEADDPVSQLHKCAFYMKDTDRMYLCLSQEKIIQFQATPCPKEPNKE
MINDGACWTIISTDKAEYQFYEGMGPVASPVTPVPIVNSLNLNGGGDVAMLELSGDNFT
PHLQVWFGDVEAETMYRCTETLLCVVPEISQFRGEWLWVRQPTQVPISLVRNDGIIYAT
GLTFTYTPEPGPRPHCNTQAEDVMRARQNNNNNNITSISNNNNSNNAGSPAAGGGLQQQ
QQQHQALPSISEVQWNSHGSGLSGPPG**YPYDVPDYA**
