# Supplementary figures and images for: Control of Neural Daughter Cell Proliferation by Multi-level Notch/Su(H)/E(spl)-HLH Signaling
Source: PLoS Genet. 2016 Apr 12;12(4):e1005984. doi: 10.1371/journal.pgen.1005984 (PMC4829154; doi:10.1371/journal.pgen.1005984)

# Supplemental Figure 1, related to Figure 1

## Notch Controls the Type I>0 Switch in NB5-6T

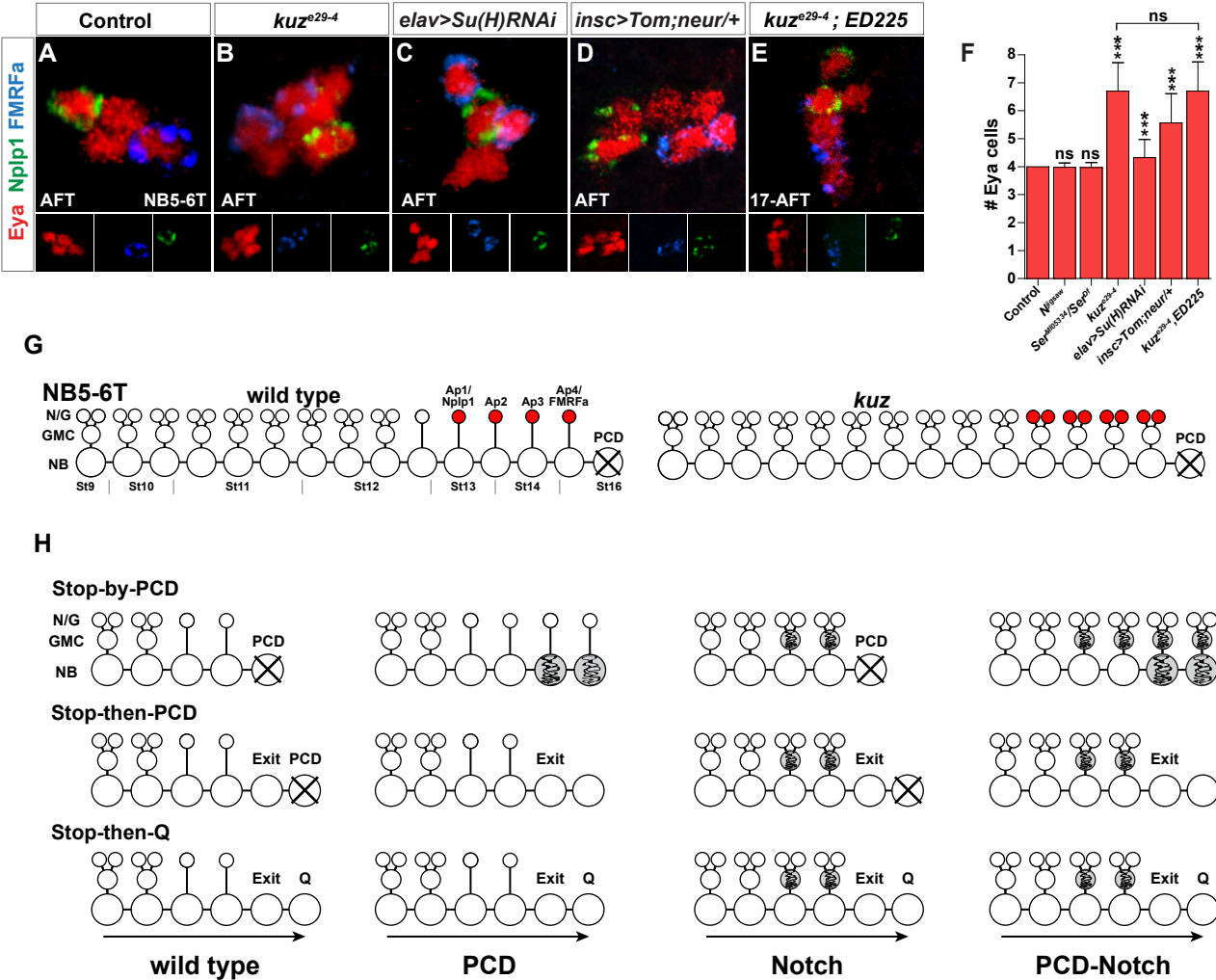

Supplement: S1 Fig — (A-E) The last-born cells in the NB5-6T lineage, the Ap neurons, are born as Type 0 cells; 4 cells in T2-T3 and 5 cells in T1; identifiable by Eya, and neuropeptides FMRFa and Nplp1. (B) kuze29-4, elav>Su(H)RNAi (C), and (D) insc>Tom;neurDf/+, frequently displayed extra Eya cells, as well as Ap1 and Ap4 duplications. (E) Although there is no apoptosis in the latter part of the NB5-6T lineage in wild type [26], previous studies demonstrated that when the Type I>0 switch is perturbed, and daughters undergo aberrant divisions, some ectopic daughters may undergo apoptosis [23]. Therefore, to reveal the full proliferation effect of kuz mutants we combined kuze29-4 with a programmed cell death (PCD) mutant (Df(3L)ED225). However, this did not result in increased Ap cell numbers beyond that observed in kuze29-4 alone. (F) Quantification of Eya cell numbers (* p≤0.05, ** p≤0.01, *** p≤0.001; n≥32 clusters; Wilcoxon signed-rank test; +/-SD). Previously, we determined that the Delta ligand was involved in this Notch event [27]. We analyzed Ser mutants and a recently generated Notch mutant, Notchjigsaw, which only affects the interaction between Notch and Ser, leaving Notch-Delta interactions un-perturbed [68]. We did however not observe any changes in Ap cell number in either mutant. (G) The NB5-6T lineage progresses with nine Type I rounds of asymmetric divisions followed by five Type 0 divisions [2]. In Notch pathway perturbations, the Type I>0 switch is perturbed leading to aberrant divisions of daughter cells. (M) Cartoons illustrating the proliferation effects in cell death (PCD), kuze29-4 (Notch) and PCD, kuz double mutants (black squiggle depicts dividing cells; Q = quiescence; exit = cell cycle exit; see Fig 2G and 2H for data). Previous studies have revealed that NBs in the VNC can stop lineage progression in three distinct ways: by programmed cell death (PCD) (“stop-by-PCD”; exemplified by NB7-3) [23,69]; by cell cycle exit followed by PCD (“stop-then-PCD”; exemplif [file pgen.1005984.s003.pdf]

# Supplemental Figure 2, related to Figure 3

## Redundancy in the *E(spl)* complex

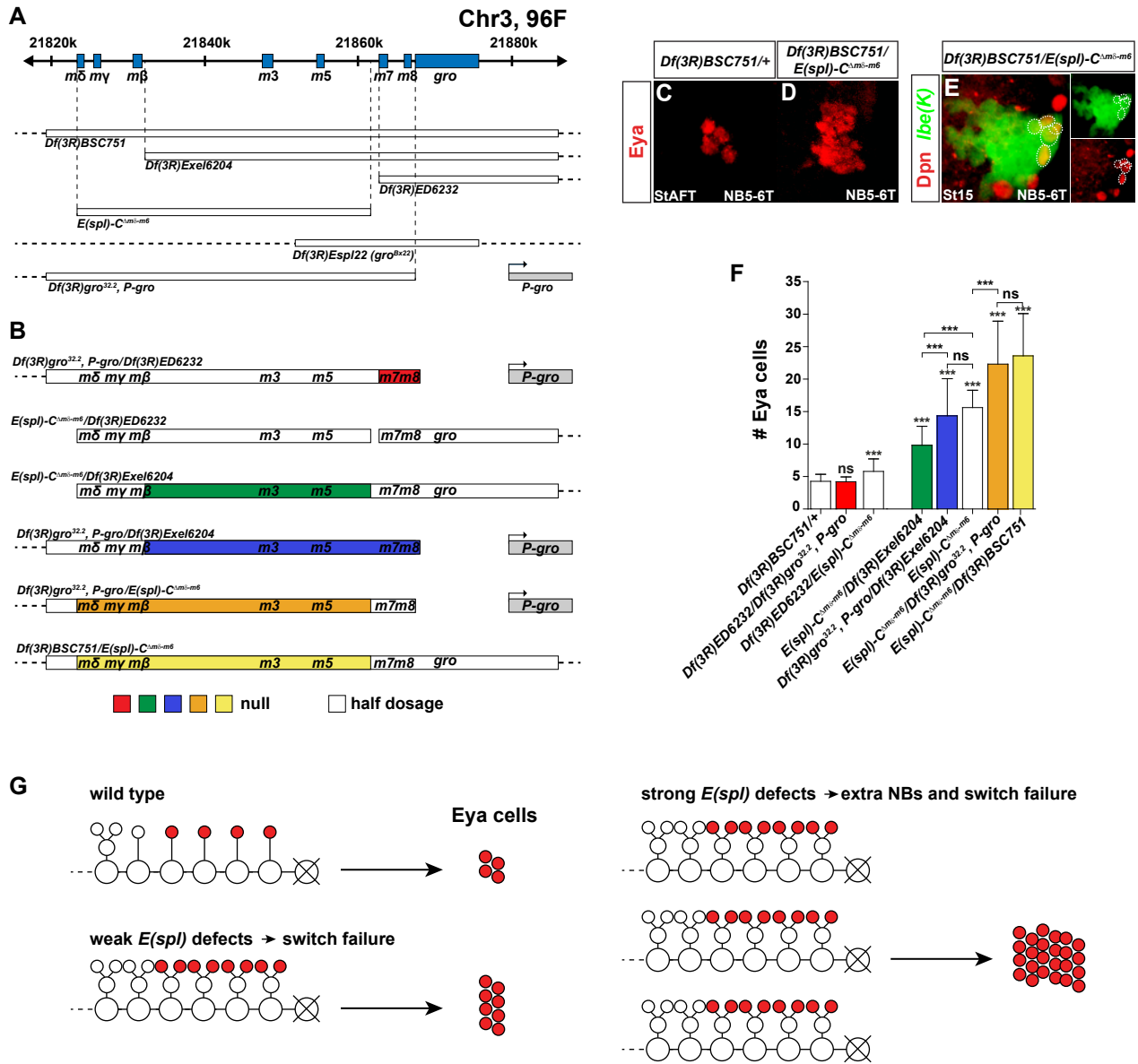

Supplement: S2 Fig — To begin addressing the role of each member of the E(spl) complex with respect to the Type I>0 switch, we first analyzed a number of deficiencies in this region, using the NB5-6T lineage and the four Ap neurons (Type 0) as readout. As anticipated, these studies revealed a complex picture of E(spl)-HLH gene involvement. The picture is further complicated by the fact that the gene encoding the E(spl)-HLH transcriptional co-repressor Groucho (Gro) is located adjacent to the E(spl) complex. (A) Schematic of the E(spl) complex, depicting only the E(spl)-HLH genes and gro; horizontal bars outline the deficiencies used. (B) Crosses made between different deficiencies. Colored overlap indicates homozygous deleted regions (null). (C) Heterozygous deletion of the entire E(spl) region, without removing gro, did not reveal any significant phenotype at stage air-filled trachea (AFT)(Df(3R)BSC751/+). Similarly, overlapping deletions removing E(spl)m7-HLH (m7) and m8 showed no significant effect (Df(3R)gro32.2, P-gro/Df(3R)ED6232; S2B, S2F). (D) In contrast, heterozygous deletion of the region, while also removing one gene copy of gro, resulted in extra Ap cells (E(spl)-CΔmδ-m6/Df(3R)ED6232; S2B, S2F). Overlapping deletions removing m3 and m5, while also removing one gene copy of gro, gave strong effects (E(spl)-CΔmδ-m6/Df(3R)Exel6204; S2B, S2F). Removal of m3, m5, as well as m7 and m8, while rescuing gro function, increased these effects (Df(3R)gro32.2, P-gro/Df(3R)Exel6204; S2B, S2F). Removal of m3, m5, as well as mδ, mγ and mβ also gave strong effects (Df(3R)gro32.2, P-gro/E(spl)-CΔmδ-m6; S2B, S2F). Further removal of gro in this background did not exacerbate this already strong effect (Df(3R)BSC751/E(spl)-CΔmδ-m6; S2B, S2D-F). (E) Expression of Dpn+ reveals extra delaminated NBs in the NB5-6T lineage, marked by lbe(K)-GFP. (F) Quantification of Eya+ cells per thoracic T2/T3 Ap cluster +/- SD, in a number of E(spl)-HLH composite deletions, at AFT. Asterisks denote significant d [file pgen.1005984.s004.pdf]

# Supplemental Figure 6, related to Figure 5 Generation of stabilized *UAS-m8*

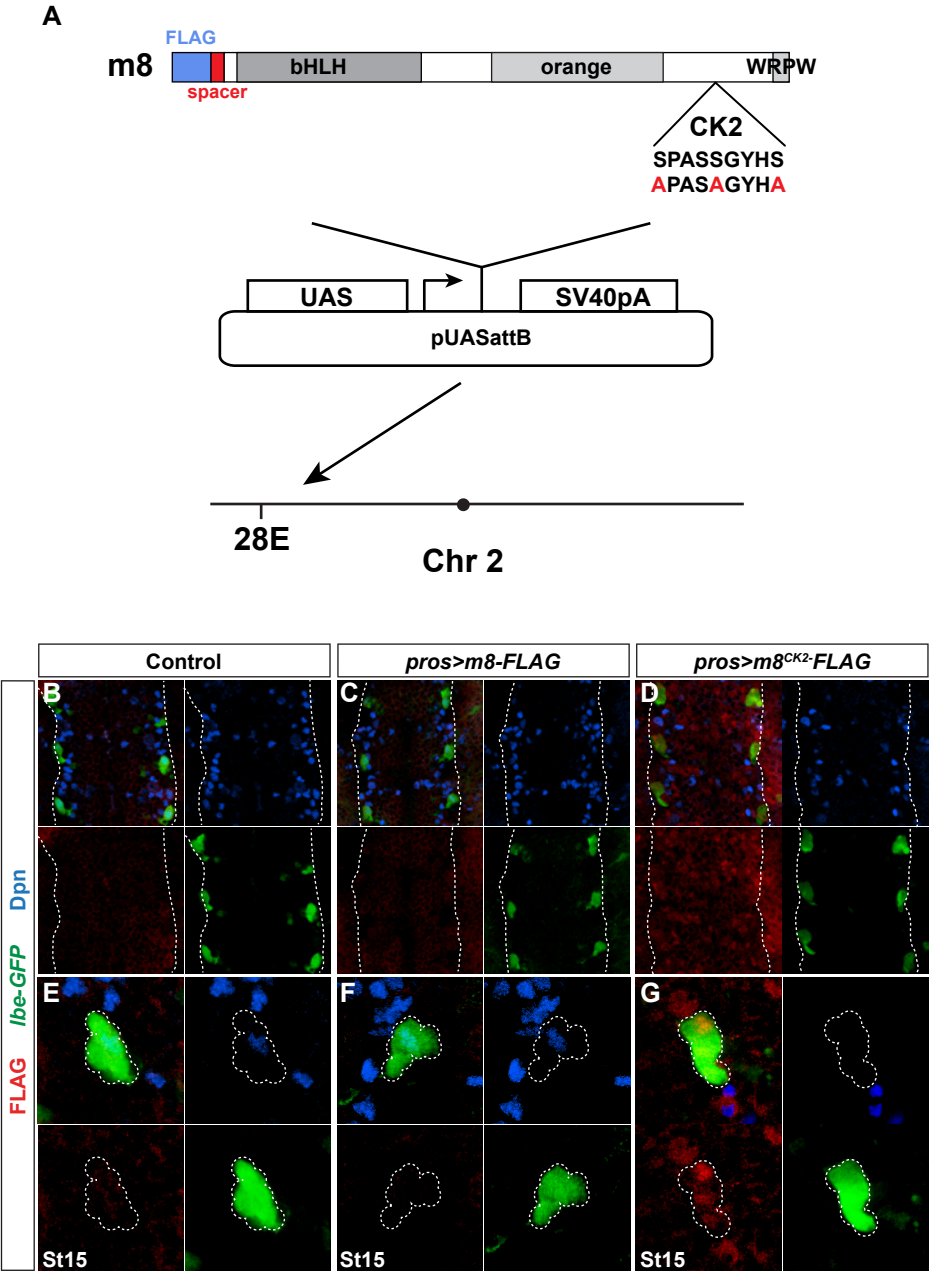

Supplement: S6 Fig — (A) New UAS-m8 constructs were generated, omitting the 5´and 3´UTR, and codon optimizing the ORF for m8. The sequence upstream the start-ATG was altered to match the Drosophila consensus and the CK2 phospho-degron was mutated. The transgene was inserted at 28E on chromosome 2. (B-G) Control and embryos expressing the novel UAS constructs, driven from pros-Gal4, detected by FLAG antibody stain. (B-D) three thoracic VNC segments; (E-G) one thoracic hemi-segment, showing NB5-6T identified by lbe(K)-GFP (St15). While expression of the m8-FLAG construct is not readily detected above control background, m8CK2-FLAG shows robust staining in the VNC and in NB5-6T. (PDF) [file pgen.1005984.s008.pdf]

# Supplemental Figure 8, related to Figure 6

## Su(H), m5 and m8 binding to *CycE* and *stg*

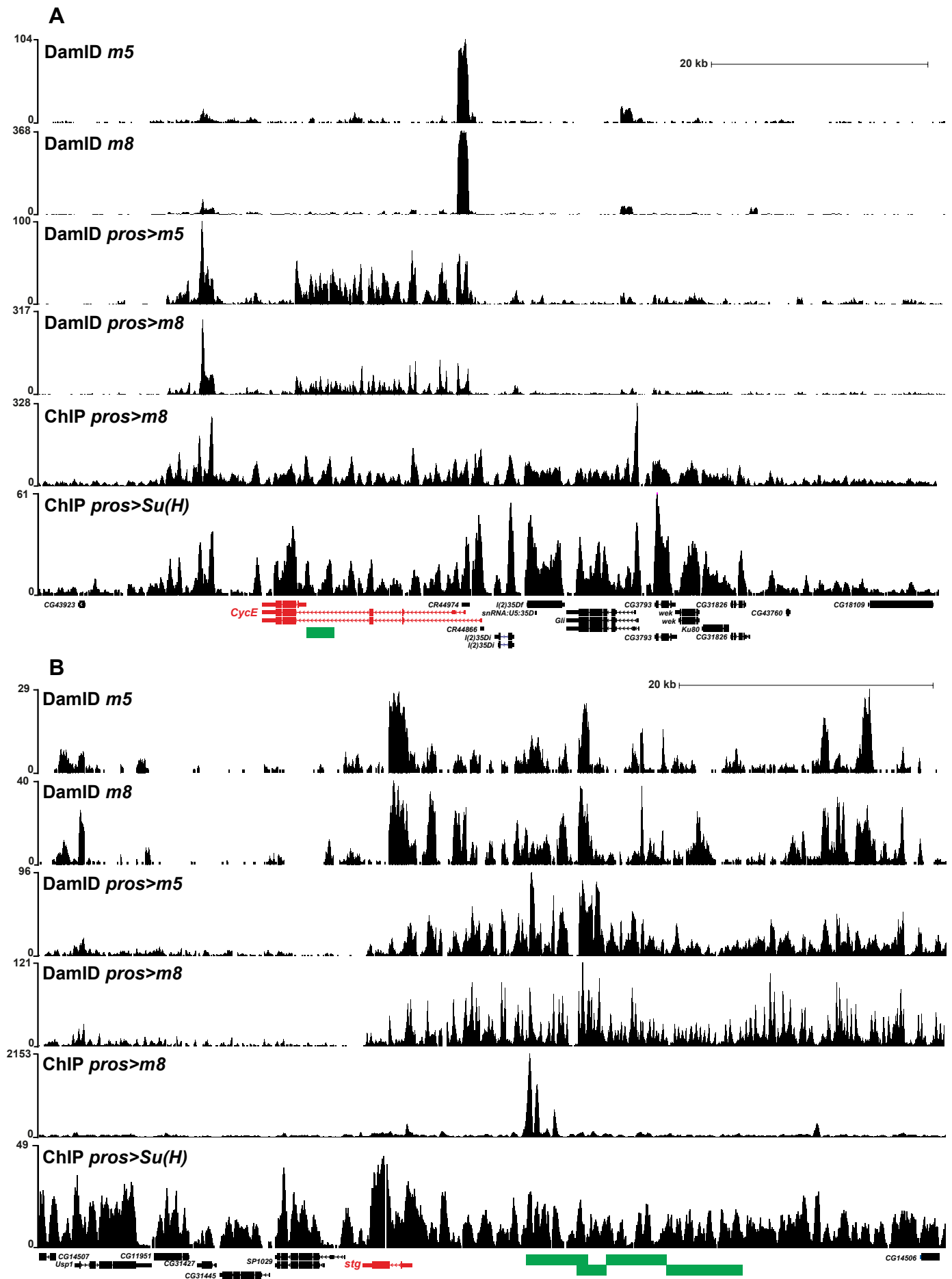

Supplement: S8 Fig — (A-B) Normalized binding profiles for ChIP of FLAG-tagged Su(H) and m8CK2, driven by pros-Gal4, as well as DamID, driven by pros-Gal4 or “un-driven”, for m5 and m8. Depicted are the CycE and stg genes. (A) Several peaks were identified on the CycE gene, and the profiles differ between the conditions used, most notably between DamID driven by pros-Gal4 or un-driven. One peak corresponds to a previously identified CycE CNS enhancer (green) [79, 80]. (B) On the stg gene, a number of peaks were detected in the upstream region. Notable difference is between m8CK2 ChIP versus m8 and m5 DamID profiles. Many peaks correspond to previously identified stg CNS enhancers (green) [81, 82]. (PDF) [file pgen.1005984.s010.pdf]
