# Supplementary material for: Control of Neural Daughter Cell Proliferation by Multi-level Notch/Su(H)/E(spl)-HLH Signaling
Source: PLoS Genet. 2016 Apr 12;12(4):e1005984. doi: 10.1371/journal.pgen.1005984 (PMC4829154; doi:10.1371/journal.pgen.1005984)

Supplemental Figure 3, related to Figure 3
Eya numbers in *E(spl)-HLH* mutants,
E(spl)m8-GFP is expressed in NBs, including NB3-3A

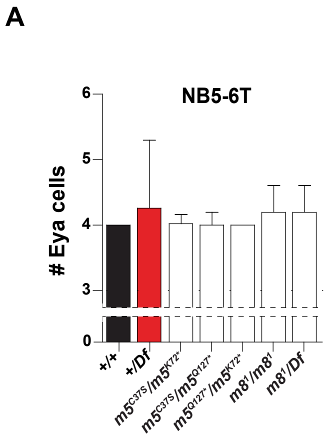

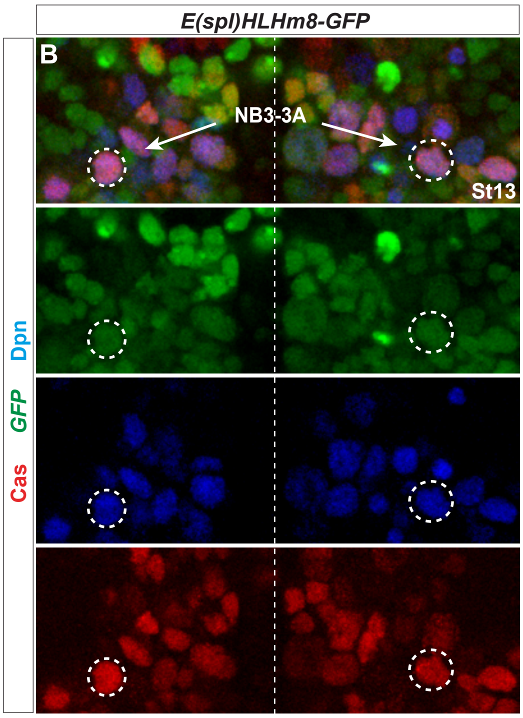

Supplement: S3 Fig — (A) Quantification of Eya cells in NB5-6T. The three TILLING alleles identified for m5 do not show significant effects when crossed to each other. This is in contrast to the results found when these m5 alleles are placed over a deletion uncovering all seven E(spl)-HLH genes (Df(3R)BSC751) (S2 Fig). The lack of effect for m8V59M in NB5-6T (Fig 4) is not likely due to that it is a hypomorphic allele, because the previously identified m81 also did not show any effect, even when placed over deficiency (Df(3R)BSC751) (* p ≤0.05, ** p≤0.01, *** p≤0.001; Kruskal-Wallis, Dunn’s posthoc; +/-SD; n≥40 segments). (B) Expression of GFP in the E(spl)HLHm8-GFP transgenic line is observed in NB3-3A, at St13. NB3-3A was identified by position, expression of Eve (not shown; out of the focal plane shown here), Dpn and Cas (known to be selectively expressed by NB3-3A at St13 [74]). (PDF) [file pgen.1005984.s005.pdf]
