# Supplementary material for: Control of Neural Daughter Cell Proliferation by Multi-level Notch/Su(H)/E(spl)-HLH Signaling
Source: PLoS Genet. 2016 Apr 12;12(4):e1005984. doi: 10.1371/journal.pgen.1005984 (PMC4829154; doi:10.1371/journal.pgen.1005984)

Supplemental Figure 4, related to Figure 4
Proneurals are not expressed in late NBs, and not affected in *kuz*

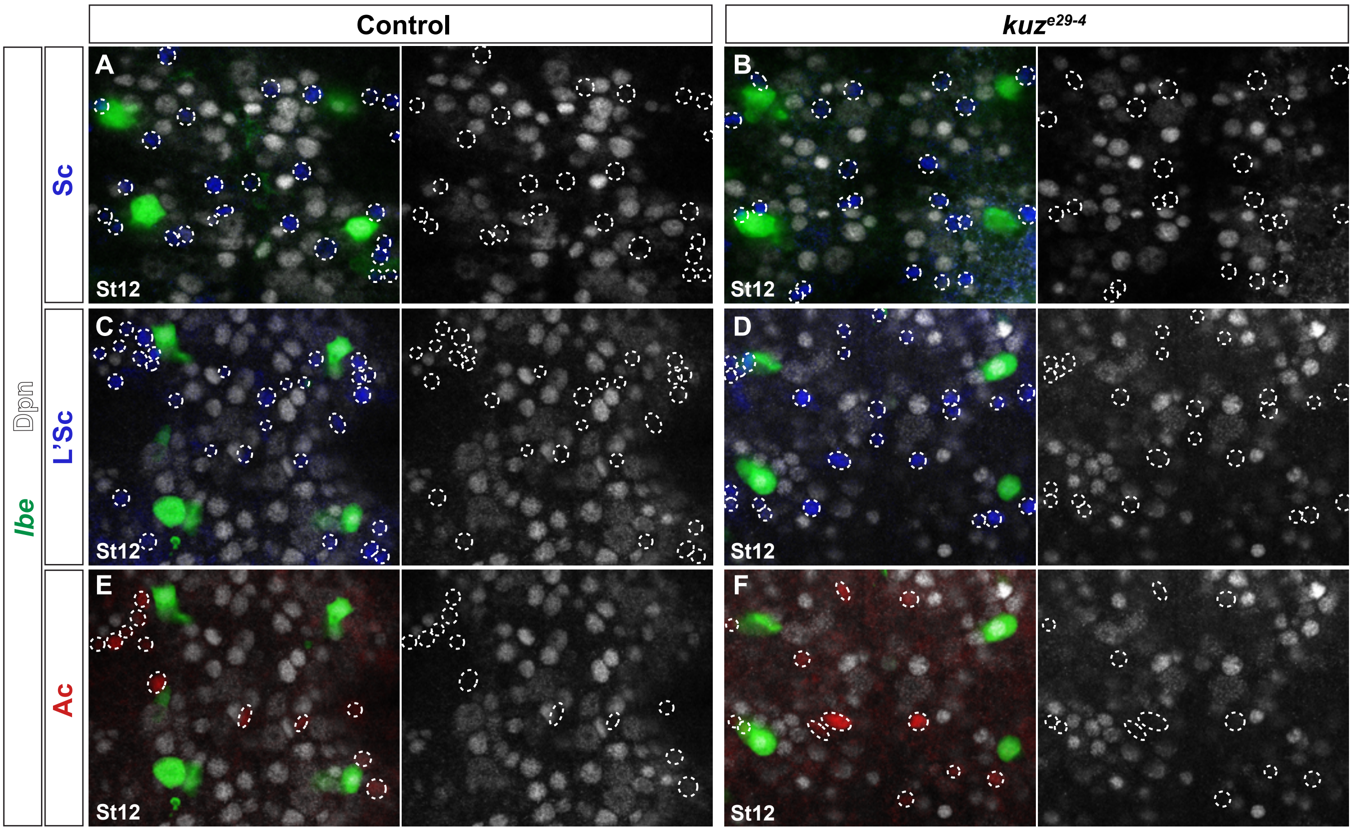

Supplement: S4 Fig — Previous studies of Notch signaling during the process of lateral inhibition demonstrated that key targets are the genes of the proneural family of bHLH transcription factors; achaete, scute and lethal-of-scute [34, 35]. Addressing the possible role of these genes in the Type I>0 switch is not trivial, first because of their genetic redundancy, and second because of their prominent role in lateral inhibition; in compound mutants many NBs fail to form during early neurogenesis [75, 76]. However, arguing against their role in NBs during the subsequent Type I>0 switch is the fact that they are known to be rapidly down-regulated immediately following NB delamination [77, 78]. (A-F) Expression of Sc, L´Sc and Ac in thoracic segments T1-T2, at StE12. The NB5-6T lineage is visualized by lbe(K)-GFP and NBs by Dpn. We stained for all three proneural proteins at St12; the time-point at which the Type I>0 switch is in progress globally. While we could detect all three proteins in a minor subset of neurons and glia, we found little if any expression in wild type NBs (A, C, E). We furthermore analyzed Ac, Sc and L-sc expression in kuze29-4, but did not observe any apparent activation of these proteins, in NBs or other cells (B, D, F). While we cannot completely rule out involvement of the proneural genes in the Type I>0 switch, their rapid down-regulation in early NBs, their apparent lack of expression in NBs at the time of the switch, and the lack of effects on proneural expression in kuz mutants, strongly argues against proneural involvement in this Notch function. (PDF) [file pgen.1005984.s006.pdf]
