# Supplementary material for: Control of Neural Daughter Cell Proliferation by Multi-level Notch/Su(H)/E(spl)-HLH Signaling
Source: PLoS Genet. 2016 Apr 12;12(4):e1005984. doi: 10.1371/journal.pgen.1005984 (PMC4829154; doi:10.1371/journal.pgen.1005984)

Supplemental Figure 5, related to Figure 5
NICD reduces daughter proliferation,
kuz interacts with *dap*

A

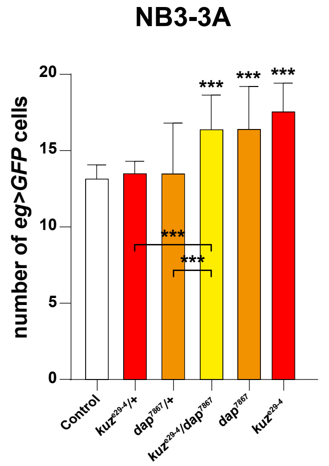

B

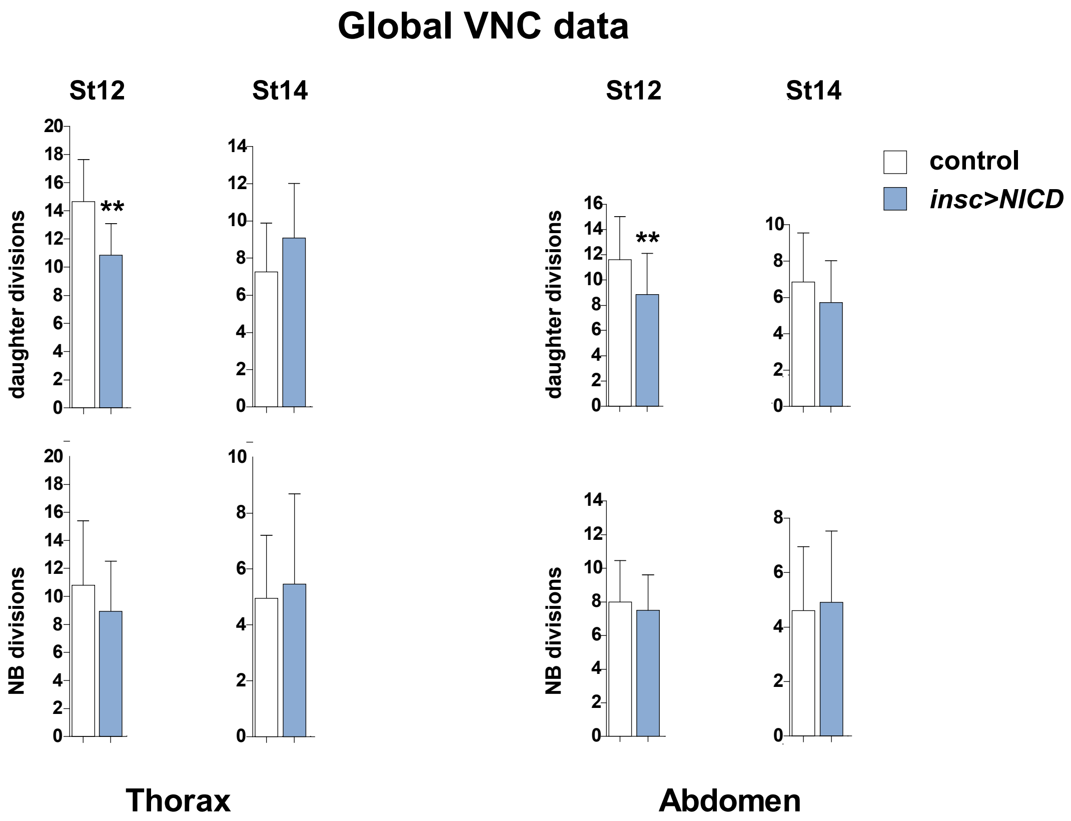

Supplement: S5 Fig — (A) Quantification of the number of NB3-3A cells, at St17 (eg-Gal4/UAS-GFP). While dap or kuz heterozygotes do not show significant increase in NB3-3A lineage cells, kuz/dap transheterozygotes show clear effects. These effects are similar to those observed in kuz or dap homozygotes (* p≤0.05, ** p≤0.01, *** p≤0.001; n≥47 lineages; ANOVA with Dunnett’s posthoc test; +/-SD). (B) Quantification of dividing NBs and daughters in the VNC in insc>NICD expression versus control; at two different developmental stages; in the thorax and abdomen (T2-T3 and A1-A2) (* p≤0.05, ** p≤0.01, *** p≤0.001; Student’s two-tailed T-test; n≥20 segments; +/-SD). Reduced daughter proliferation is observed at St12, in both thorax and abdomen, while NBs are unaffected. (PDF) [file pgen.1005984.s007.pdf]
