# Supplementary material for: Control of Neural Daughter Cell Proliferation by Multi-level Notch/Su(H)/E(spl)-HLH Signaling
Source: PLoS Genet. 2016 Apr 12;12(4):e1005984. doi: 10.1371/journal.pgen.1005984 (PMC4829154; doi:10.1371/journal.pgen.1005984)

Supplemental Figure 7, related to Figure 5
Stg expression levels in *pros>m8Ck2* and *kuz;*
number of cells in NB5-6T lineage in *pros>m8Ck2*

A

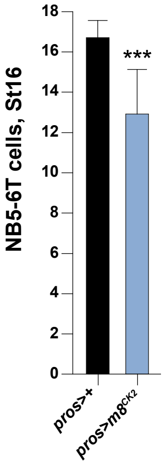

B

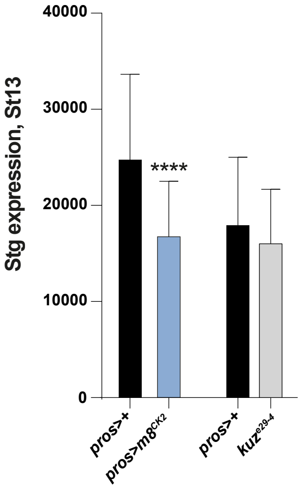

Supplement: S7 Fig — (A) Expression of m8CK2, driven by pros-Gal4, results in significantly reduced cell numbers in the NB5-6T lineage (* p≤0.05, ** p≤0.01, *** p≤0.001; +/-SD; Student’s two-tailed T-test; n≥ 24 lineages). (B) Expression of m8CK2, driven by pros-Gal4, results in reduced levels of Stg expression. In kuze29-4 we did not observe significant changes in Stg (* p≤0.05, ** p≤0.01, *** p≤0.001; +/-SD; Student’s two-tailed T-test; n≥26 NBs). (PDF) [file pgen.1005984.s009.pdf]
