# Supplementary material for: Control of Neural Daughter Cell Proliferation by Multi-level Notch/Su(H)/E(spl)-HLH Signaling
Source: PLoS Genet. 2016 Apr 12;12(4):e1005984. doi: 10.1371/journal.pgen.1005984 (PMC4829154; doi:10.1371/journal.pgen.1005984)
Supplement: S1 Table — Primer pairs and PCR fragment size used for the TILLING of the seven E(spl)-HLH genes. The mutated nucleotide and corresponding amino acid mutation is also shown, as well as the stock numbers from the Stowers collection and the Bloomington stock numbers. (PDF) [file pgen.1005984.s011.pdf]

Supplemental Table 1, related to Figure 3. Identification of mutations in the *E(spl)* genes by Fly-TILL

Gene	PCR primer pairs	# mutations identified	Mutation	AA change	PCR frag. (bp)	Stowers Stock #	Bloomington Stock #
m8	m8-539 ttagccgtgcgagcgaaaga	8	G343A	V59M	911	SH_1251	61149
	m8-1450 tcccctcccaacatcccaataaag						
m7	m7-314 gctccgcaggtggtggtt	12	G268A	G86E	1011	SH_1228	61150
	m7-1325 agacttctgctcgattgattta		G249A	E80K		SH_1098	61151
			T322A	V104E		SH_0477	61152
m5	m5-1334 cgatcatcaggcgggggaggta	12	A278T	K72*	1364	SH_0665	61153
	m5-2698 gctgcggctgctgcgggtat		C443T	Q127*		SH_0507	61154
			T173A	C37S		SH_0357	61155
			C582T	T173I		SH_0738	61156
m3	m3-2294 gcaacgcacgcgcaacaaactc	5	G144A	E4K	1383	SH_0625	61157
	m3-3677 tggcggactgggacaagaatgaac						
m γ	gamma-4949 tgcgggcaggtgagcgagtc	7	G280A	G50D	1344	SH_0119	61158
	gamma-6293 ttgcgtttctagcgtttccgttct		T437A	N102K		SH_1107	61159
m δ	delta-4356 acggagtggggagagttca	8	G637T	E161*	1406	SH_0313	61160
	delta-2950 gcgtggtggcagttagg		T323A	L56Q		SH_0657	61161
m β	beta-2636 cgccggcgcaaaacttcc	14	G445A	E47K	1377	SH_0839	61162
	beta-4013 tgtcccgctcgcactcactgtc		T621A	H105Q		SH_0732	61163
	Total number of mutations identified:	66					
